# Pax7 remodels the chromatin landscape in skeletal muscle stem cells

**DOI:** 10.1371/journal.pone.0176190

**Published:** 2017-04-25

**Authors:** Karin C. Lilja, Nan Zhang, Alessandro Magli, Volkan Gunduz, Christopher J. Bowman, Robert W. Arpke, Radbod Darabi, Michael Kyba, Rita Perlingeiro, Brian D. Dynlacht

**Affiliations:** 1 Department of Pathology, New York University Cancer Institute, New York University School of Medicine, New York, New York, United States of America; 2 Lillehei Heart Institute, University of Minnesota, Minneapolis, MN, United States of America; 3 Department of Pediatrics, University of Minnesota, Minneapolis, MN, United States of America; 4 The University of Texas Health Science Center at Houston, Houston, TX, United States of America; University of Minnesota Medical Center, UNITED STATES

## Abstract

Pluripotent stem cells (PSC) hold great promise for the treatment of human skeletal muscle diseases. However, it remains challenging to convert PSC to skeletal muscle cells, and the mechanisms by which the master regulatory transcription factor, Pax7, promotes muscle stem (satellite) cell identity are not yet understood. We have taken advantage of PSC-derived skeletal muscle precursor cells (iPax7), wherein the induced expression of Pax7 robustly initiates the muscle program and enables the *in vitro* generation of precursors that seed the satellite cell compartment upon transplantation. Remarkably, we found that chromatin accessibility in myogenic precursors pre-figures subsequent activation of myogenic differentiation genes. We also found that Pax7 binding is generally restricted to euchromatic regions and excluded from H3K27 tri-methylated regions in muscle cells, suggesting that recruitment of this factor is circumscribed by chromatin state. Further, we show that Pax7 binding induces dramatic, localized remodeling of chromatin characterized by the acquisition of histone marks associated with enhancer activity and induction of chromatin accessibility in both muscle precursors and lineage-committed myoblasts. Conversely, removal of Pax7 leads to rapid reversal of these features on a subset of enhancers. Interestingly, another cluster of Pax7 binding sites is associated with a durably accessible and remodeled chromatin state after removal of Pax7, and persistent enhancer accessibility is associated with subsequent, proximal binding by the muscle regulatory factors, MyoD1 and myogenin. Our studies provide new insights into the epigenetic landscape of skeletal muscle stem cells and precursors and the role of Pax7 in satellite cell specification.

## Introduction

A major unexplored area in muscle biology pertains to the molecular mechanisms that drive specification of muscle stem cells, also known as satellite cells (SC). Understanding genome-wide networks that underlie muscle stem cell specification has been met with several significant technical challenges. First, SC represent a low-abundance population within muscle tissue. Once removed from its niche, satellite cells are activated and differentiate, and they can no longer repopulate the stem cell niche. Second, it is technically challenging to obtain skeletal muscle cells through embryonic stem cell (ESC) differentiation, since transcription factors that specify the muscle lineage within paraxial mesoderm, Pax3 and Pax7, are not sufficiently expressed during the in vitro differentiation of ES cells [1, 2]. Although recent studies have shown that ESCs can be differentiated into skeletal muscle through treatment with cocktails of small molecule inhibitors and growth factors [3–5], these treatments often lead to heterogeneity in the resulting populations.

Furthermore, although transcriptional and epigenetic mechanisms that underlie muscle differentiation or “stemness” in ESCs have been intensively studied, much less is known about cues that specify skeletal muscle stem cell identity. Since one epigenetic mark, H3K27 tri-methylation (H3K27me3), has been shown to accumulate in aging SC—thus suggesting a potential role in the sarcopenia-related functional decline of SC [6, 7]—it will be essential to understand the dynamics of this repressive modification as well as other signatures associated with SC identity and regeneration. To bridge this gap, engineered ES cell lines that inducibly express Pax7 or Pax3 (termed iPax7 or iPax3 ES cells) and form myogenic precursors have recently been developed [1, 8, 9]. Induction of Pax7 expression during embryoid body formation from ESC allows for the generation of large numbers of skeletal myogenic precursors (hereafter referred to as iPax7 cells). Another major advantage of these cells is that they can be propagated in the presence of inducer, setting them apart from SC, which tend to spontaneously differentiate after isolation from myofibers. Importantly, both human and mouse iPax7 cells have been shown to repopulate the satellite cell niche in damaged muscle upon transplantation into immune-compromised and dystrophic mice and to ameliorate the impact of muscle wasting in a muscular dystrophy (*mdx*) model [8, 9]. These studies support the idea that iPax7 cells can faithfully mimic bona fide muscle precursors *in vivo*, offering the possibility of future therapeutic applications.

In an effort to understand the role of Pax7 in specifying skeletal muscle stem cell identity, we took advantage of iPax7 muscle precursors to examine the transcriptomic and epigenetic landscape governed by this master regulator. We found that myogenic precursors exhibited accessible chromatin at genes that will be subsequently activated during myogenic differentiation, suggesting an unforeseen, inherent plasticity. We also found that Pax7 exhibits an ability to generate active chromatin modifications and to create regions of open chromatin at enhancers. Interestingly, active features on one cluster of Pax7 bound regions diminish after Pax7 removal, whereas a subset of sites persists after Pax7 expression wanes. These latter sites could be maintained as enhancers, since they remain accessible and subsequently recruit muscle regulatory factors, MyoD1 and Myogenin, in a differentiation-dependent manner. Importantly, these latter sites are rendered accessible upon commitment to the myogenic fate, since mouse ES cells do not display accessibility at these sites. These studies provide a valuable resource for understanding the epigenetic landscape of myogenic precursors and allow for the mechanistic dissection of Pax7, which is able to remodel chromatin in muscle stem cells.

## Results and discussion

### Characterization of myogenic precursors

**Fig 1:**
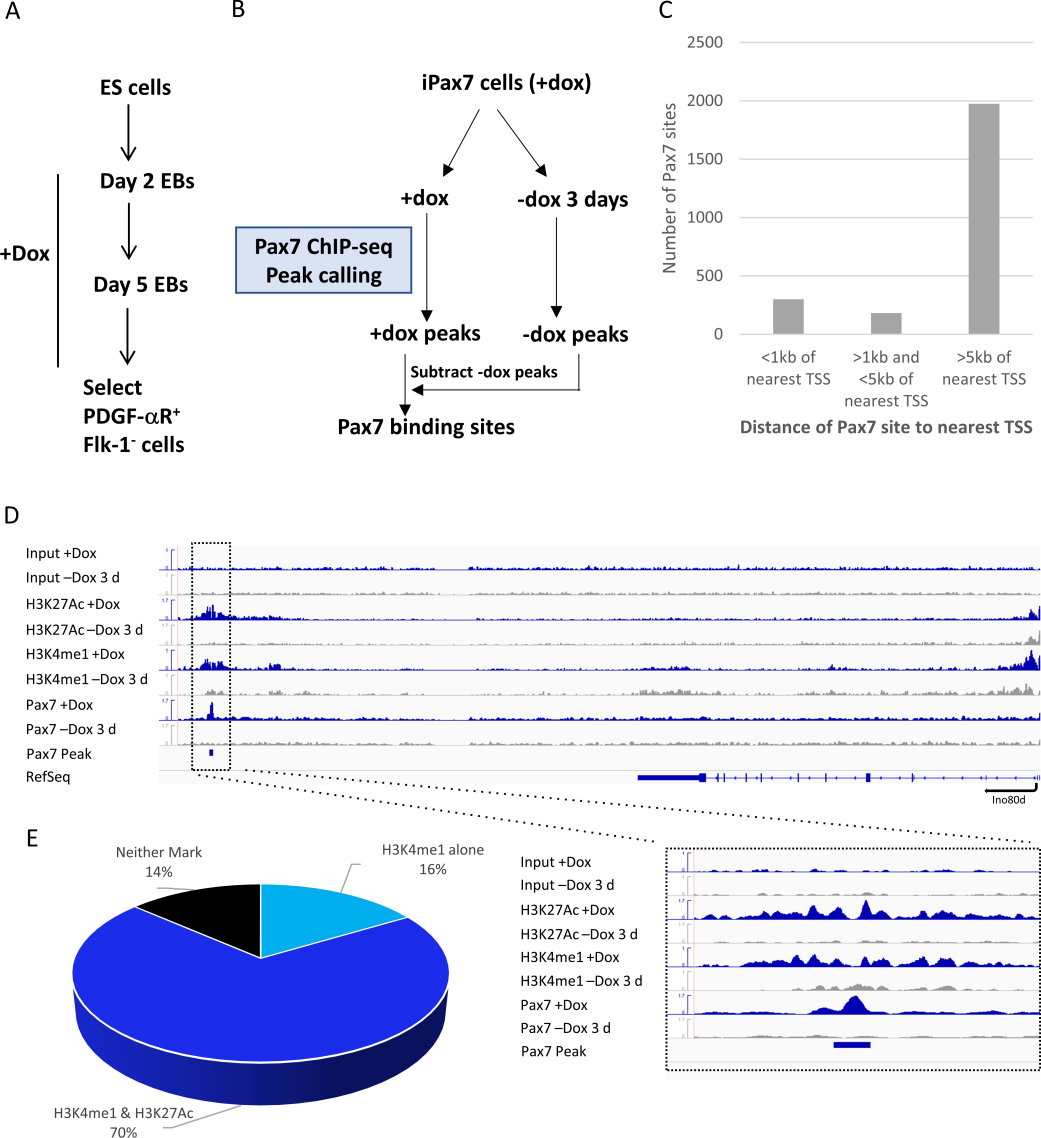
Identification and characterization of Pax7 bound regions in iPax7 cells. (**A**) Schematic showing iPax7 cell isolation after inducing Pax7 expression with doxycycline (Dox) during embryoid body (EB) formation and sorting the PDGFαR+Flk-1- fraction (Darabi et al., 2011). (**B**) Pax7 ChIP-seq was performed using iPax7 cells grown in the presence of Dox or after removal of Dox for 3d. Regions that were more highly enriched in the presence of Dox were identified as Pax7 binding sites. (**C**) The majority of 2455 Pax7 binding sites are > 5 kb from the nearest transcription start site (TSS), whereas 300 binding sites were found at promoter regions within 1 kb of the nearest TSS. (**D**) Representative read density profiles for Pax7, H3K4me1, and H3K27Ac enrichment, and input in +Dox (blue) and -Dox (grey) conditions. Enlarged region shows enhancer-associated histone modifications (H3K4me1 and H3K27Ac) are enriched at distal Pax7 binding sites in +Dox versus -Dox conditions. The *y*-axis corresponds to normalized read densities. (**E**) Pie chart depicting percentages of distal (>1 kb from TSS) Pax7 enriched regions that overlap with H3K4me1, H3K27Ac, both marks, or neither histone modification.

In an effort to examine the chromatin environment of myogenic precursors and investigate potential remodeling during skeletal muscle differentiation, we took advantage of the mouse iPax7 ESC line, which inducibly expresses Pax7 in the presence of doxycycline (Dox)[9, 10]. Muscle precursor cells were purified on day 5 of embryoid body differentiation based on the expression of PDGFαR and lack of Flk-1 (PDGFαR^+^Flk-1^-^), as previously described (Fig 1A; S1 Fig)[9, 10]. As expected, Pax7 was induced in these cells in the presence of Dox, whereas its levels declined precipitously within 12h after removal of the inducer and diminished to very low levels within 3d (S1B Fig). We also examined transcript and protein levels for muscle regulatory factors (MRFs; Myf5 and Myogenin [Myog]) and terminal differentiation markers (myosin heavy chain, MHC) in cells grown in Dox and three days after its withdrawal and found that down-regulation of Myf5 coincided with the induction of Myog and subsequent expression of MHC (S1 Fig). Accordingly, iPax7 cells proliferated in the presence of Dox, and removal of this inducer for three days led to formation of elongated cells that subsequently fused to form myotubes in the presence of differentiation medium containing horse serum (S1A Fig; [9]).

In an effort to understand regulatory programs controlled by Pax7, we examined the transcriptome of iPax7 muscle precursor cells by performing high throughput sequencing (RNA-seq) on Dox-treated populations and compared this profile with iPax7 cells 3 days after Dox withdrawal, since Pax7 was expressed at extremely low levels in the latter condition (S1B Fig). We also compared the transcriptome of iPax7 cells with that of satellite cells isolated from mouse skeletal muscle tissue using a Pax7-ZsGreen reporter strain [11]. These studies suggested a stronger correlation between expression profiles of satellite cells and iPax7 cells grown in Dox versus cells lacking this inducer (S2A Fig). This finding is consistent with the idea that precursors expressing Pax7 (satellite and Dox-treated iPax7 muscle precursor cells) were more highly related to one another, despite the fact that both iPax7 populations (+ and -Dox) were derived from the same origin and differed only with respect to the presence/absence of inducer.

In an effort to identify a signature that typifies myogenic precursors and satellite cells, we focused on genes that were transcribed in muscle-derived satellite cells and were more highly expressed (by ≥2-fold) in iPax7 cells grown with Dox versus without Dox. This analysis led to the identification of 2009 genes that were down-regulated upon loss of Dox, whereas 1795 genes were up-regulated after Dox removal (S1 Table). Classification using Gene Ontology (GO) indicated that genes related to cell cycle, DNA repair, migration, and cell adhesion were down-regulated after removal of Dox. These findings are consistent with the transition from a proliferative to a more highly differentiated state upon loss of Pax7 [9]. The group of cell adhesion genes is especially interesting, as it contained multiple integrin genes and cell surface markers (*Kitl*, *Lama2*, *CD44*, *CD34*, *CXCR4*, *Vcam1*), which may be relevant for progenitor populations, particularly satellite cells—which occupy a unique location between the basal lamina and the myofiber plasma membrane—and, notably, several of them represent Pax7 targets (see below; S2 Table). In addition, >100 transcription factor genes, including *Myf5*, *Dmrt2*, *Lef1*, *Runx2*, *Tcf7l2*, *Tshz3*, and *CTCF*, were also highly enriched in this gene set. Many of these factors play roles in muscle development. As examples, *Myf5* is a known target of Pax7, and *Dmrt2* was shown to be a target of Pax3 [12, 13]. These findings suggest a broad transcription factor network acting downstream of Pax7. Conversely, genes related to myogenic differentiation and myofiber structure and function were up-regulated after Dox withdrawal (S2B Fig) [14]. We also performed ChIP-seq for active (H3K4me3) and repressive (H3K27me3) modifications and queried their enrichment at promoter regions in Dox-treated iPax7 cells then globally compared these data to analogous data obtained from activated satellite cells (ASC; [6]) and C2C12 myoblasts and myotubes [14](S2C Fig). We found a striking overall concordance between iPax7 cells and activated satellite cells, and this correlation dramatically decreased in comparisons with myoblasts or myobtubes. Thus, our myogenic precursors exhibited a striking resemblance to activated satellite cells obtained from skeletal muscle, and altogether, these studies suggest the utility of iPax7 cells as a valuable resource for identifying additional SC markers and examining the role of Pax7 in controlling satellite cell identity and expression signatures. We note that quiescent satellite cells may have related, but slightly different, expression and epigenetic signatures that reflect their non-proliferative state. Thus, our system is likely to model many aspects of native, activated satellite cells, but other aspects of quiescent cells, including expression signatures associated with compacted chromatin and other components of the niche, such as cell-cell interactions, may not be captured by iPax7 cells.

### Identification of Pax7 binding sites in myogenic precursors

In the remainder of our study, we focused on the role of Pax7 as a regulator of the chromatin landscape in muscle progenitors. The low abundance of satellite cells *in vivo* poses significant challenges for obtaining sufficient material to identify native Pax7 binding sites. To bypass this obstacle, chromatin immunoprecipitation (ChIP) coupled with high throughput sequencing (ChIP-seq) has been performed on primary myoblasts over-expressing tagged Pax7 [15]. However, Pax7 was highly expressed (>20-fold) in this setting [15], leading to potential over-estimation of binding sites. Moreover, since Pax7 was introduced in a population (primary myoblasts) lacking the capacity to repopulate the SC niche *in vivo*, we anticipated that additional targets of Pax7 relating to stem cell function were yet to be discovered. To overcome this limitation, we used iPax7 muscle precursor cells induced to express Pax7, wherein ~2-fold higher levels of Pax7 were expressed by RNA-seq as compared to muscle-derived satellite cells (S1 Table). We examined Pax7 recruitment using a well-characterized monoclonal antibody and showed that Pax7 was specifically enriched on multiple genomic regions in the presence, but not the absence, of Dox, including several known targets, such as *Myf5* enhancers (S3A Fig).

Next, we performed ChIP-seq on Pax7 cells grown in the presence or absence of Dox and identified enriched genomic regions after peak-calling [14]. In an effort to obtain a high-confidence set of Pax7 targets, we subtracted enriched regions observed in cells grown without Dox from those obtained in Dox-treated cells, and this resulted in the identification of 2455 Pax7 bound regions (Fig 1B). It is likely that this approach will lead to an under-estimation of the number of Pax7 binding sites, since Pax7 may persist on a subset of sites in the absence of Dox, and these sites may not be included in our list. We also note that ~50% of our targets overlapped with genomic regions identified using tandemly-tagged Pax7 ectopically expressed in primary myoblasts, although the latter study identified a substantially larger number of possible targets, perhaps owing to vastly elevated Pax7 levels, expression of this protein in a distinct population (myoblasts), use of a different antibody for ChIP, or a combination of all of these factors (S3B Fig; [15]). More importantly, we identified 1222 previously undisclosed Pax7 targets in our myogenic precursor population, iPax7, that is capable of repopulating the in vivo satellite cell niche, vastly expanding the repertoire of Pax7 binding sites. Using the MEME DNA sequence motif discovery algorithm, we showed that the reported Pax7 paired and homeobox recognition motifs were enriched among regions bound by Pax7 in this precursor population (S3B Fig).

We performed GO analysis after assigning each binding site to its nearest neighboring gene and found that Pax7 target gene categories included regulation of transcription and proliferation, muscle development, and cell adhesion and migration (S3C Fig). We further analyzed our compendium of Pax7 binding sites and found that the vast majority (1974/2455 or 80%) of these sites were distal (>5 kb) to the nearest gene, whereas only 300 sites were in promoter-proximal locations within 1 kb of the nearest transcription start site (TSS), consistent with a previous analysis in myoblasts (Fig 1C)[15]. We found that the median distance from a Pax7 binding site to the nearest TSS was 41 kb (mean distance of 99 kb), suggesting that this factor functions primarily through long-distance enhancer interactions with its target genes.

### Pax7 alters chromatin modifications in muscle precursors

**Fig 2:**
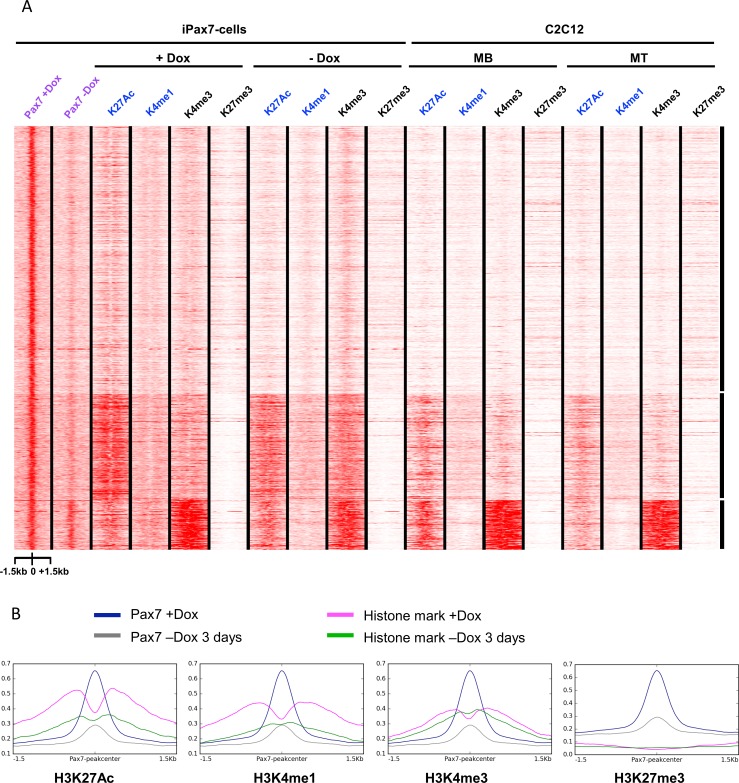
Pax7 binding demarcates and maintains potentially active enhancers. (**A**) Heatmap generated using *k*-means clustering analysis indicates that Pax7 binding sites are preferentially and globally enriched for histone modifications associated with enhancer marks (H3K27Ac and H3K4me1) in iPax7 cells treated with Dox. Enhancer marks were reduced upon removal of Pax7, indicating that maintenance of a significant number of enhancers could depend on Pax7 binding. Only a fraction of Pax7 binding regions enriched for the enhancer signature in Dox-treated cells were also enriched in C2C12 myoblasts and myotubes. ChIP-seq data were normalized for each antibody and plotted for regions 1.5 kb upstream and downstream of the center of Pax7 binding sites. MB and MT, myoblasts and myotubes, respectively. (**B**) Metagene analysis of average signal for ChIP-seq data displayed in panel A, plotted for regions 1.5 kb upstream and downstream of the center of Pax7 binding sites. Levels of indicated enhancer marks were either reduced or unchanged after removal of Pax7.

In an effort to further characterize the impact of Pax7 on the epigenetic landscape of muscle precursors, we performed a genome-wide examination of key regulatory histone modifications in iPax7 cells. Since our goal was to focus on Pax7-dependent changes in chromatin at regulatory elements, we used ChIP-seq to examine key modifications associated with promoters (e.g., enrichment of H3K4me3 and H3K27me3) and enhancers (e.g., H3K4me1 and H3K27ac enrichment) in iPax7 cells in the presence and absence of Dox. Remarkably, we found that expression of Pax7 had a dramatic, genome-wide impact on active histone marks proximal to its binding sites. In particular, we found that removal of Pax7 led to localized reductions in H3K4me1 and H3K27ac near Pax7 bound sites for many target genes, without resulting in significant changes in H3K27me3 (Figs 1D, 1E and 2). These genomic regions potentially demarcate enhancers active in myogenic precursors expressing Pax7. Regions exhibiting a “bimodal” nucleosomal configuration, which typifies active chromatin, were particularly noteworthy in the Dox-induced condition [16], and these differences were more evident when we subjected these data to metagene analysis (Fig 2A and 2B). The loss of bimodal signatures for H3K4me1 and H3K27ac upon removal of Pax7 was significant, in sharp contrast with H3K4me3 and H3K27me3, which showed little or no change.

**Fig 3:**
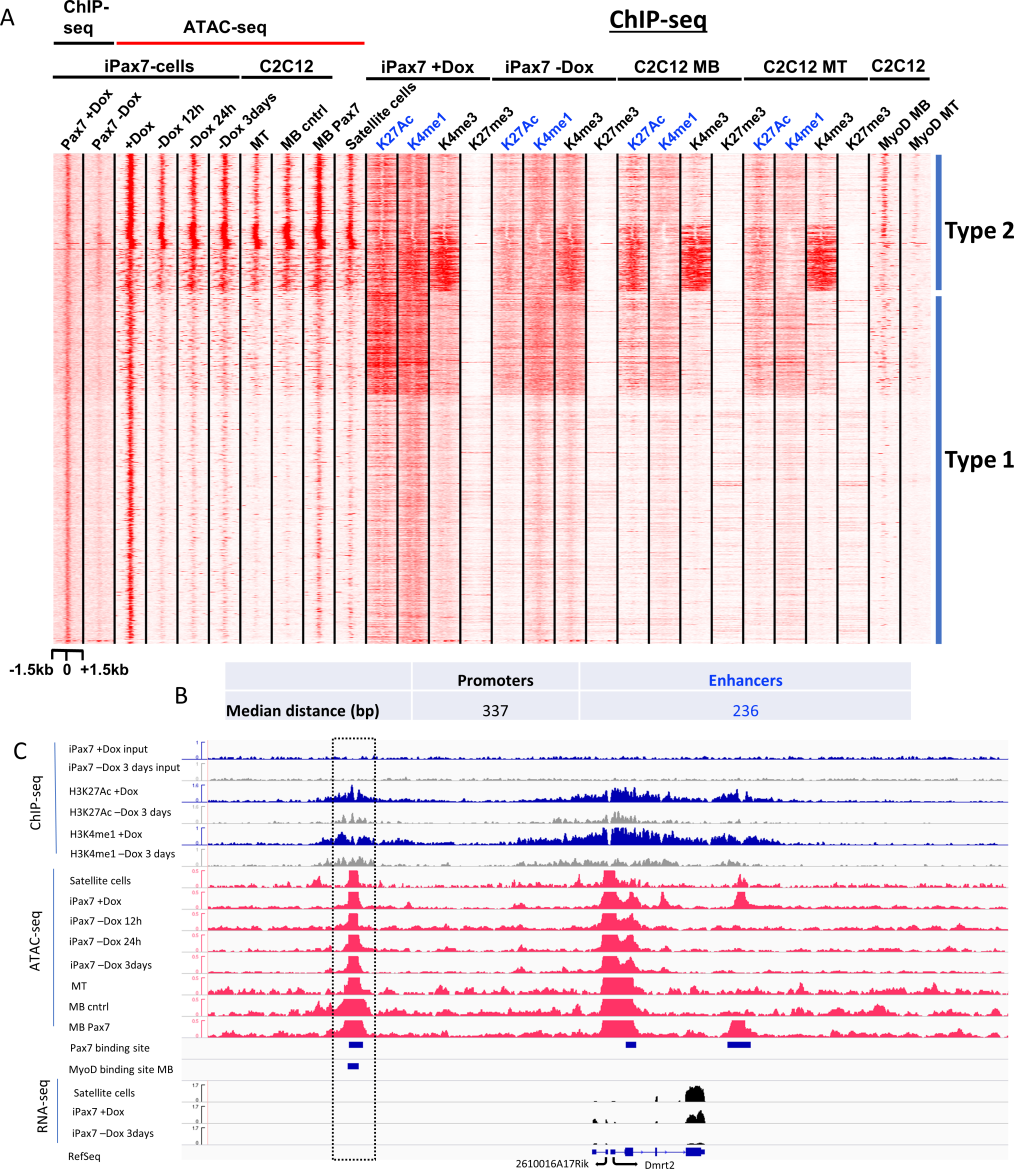
Pax7 is essential to maintain open chromatin and enhancer signatures at a subset of target genes. (**A**) Heatmap of ATAC-seq and ChIP-seq data highlight two distinct types of Pax7 binding sites, as indicated. Type 2 sites are accessible in satellite cells, iPax7 cells, and throughout myogenesis, and these sites are proximal to regions bound by MyoD in myoblasts (MB). Type 1 sites exhibit accessibility and enhancer signatures that strictly depend on Pax7 binding. (**B**) Pax7 and MyoD binding sites are proximal to one another. The center-to-center distances between MyoD- and Pax7 ChIP-seq peaks were analyzed at promoters and enhancers. (**C**) Illustrative read density profiles for ATAC-seq, ChIP-seq, and RNA-seq data corresponding to a Type 2 Pax7 binding site. Highlighted region indicates the proximity of Pax7 and MyoD binding sites at a potential enhancer that is accessible in satellite cells, iPax7 cells, and throughout myogenesis. Promoter regions bound initially by Pax7 are accessible throughout myogenesis in the absence of Pax7, as indicated by times after Dox withdrawal. Pax7-dependent Type 1 enhancers (encompassing right-most Pax7 binding site) are accessible only in the presence of Pax7, and these regions lose both enhancer marks (H3K27Ac and H3K4me1) upon removal of Pax7. MB and MT, myoblasts and myotubes, respectively. MB cntrl and MB Pax7 indicate C2C12 cell lines without or with ectopic expression of Flag-Pax7, respectively.

Importantly, we also identified two groups of Pax7-dependent enhancers: one cluster lost H3K27ac within 3d after Dox removal, and H3K4me1 was concomitantly lost or diminished. A second group maintained both enhancer marks several days after removal of Pax7, and these marks were also observed in iPax7 cells differentiated to myotubes, as well as C2C12 myoblasts and myotubes (Figs 2 and 3). Loss of Pax7 expression generally led to less dramatic alterations in H3K4me3 than either H3K4me1 or H3K27ac (Fig 2B). These findings indicate that Pax7 is able to alter chromatin modifications in myogenic precursors, and they are consistent with the conclusion that Pax7 acts at distal enhancers to effect its functions.

### Pax7 binding induces chromatin accessibility in muscle precursors

Next, we further investigated whether Pax7 binding sites displayed other features of enhancer elements, such as chromatin accessibility, and whether such accessibility was strictly dependent on Pax7 expression. We used an assay for transposase-accessible chromatin (ATAC-seq; [17]) to investigate an association between Pax7 binding and chromatin accessibility. We performed ATAC-seq on iPax7 cells grown in the presence of Dox and on populations 12h, 24h, and 3d after removal of Dox. We also compared regions of open chromatin in each of these populations to those in satellite cells isolated from mouse hind limb skeletal muscles and C2C12 myoblasts and myotubes. Using principal component analysis (PCA), we found that iPax7 populations clustered near one another and were easily separable from C2C12 myoblasts and myotubes, which do not express Pax7 (S4A Fig). Remarkably, however, when we restricted our analysis to accessible regions that overlapped Pax7 binding sites, we found that we could cleanly separate populations of cells expressing Pax7 (+Dox) from those wherein this transcription factor had been removed for even a short duration (12h -Dox) (S4B Fig). This is consistent with our comparisons of accessibility of Pax7 bound sites in muscle precursors and myoblasts with tissue-derived SC. Indeed, when we included SC in this analysis, we found that all three cell types expressing Pax7 (satellite cells, iPax7 +Dox, and C2C12 myoblasts expressing Pax7; see below) were clearly separable from any population lacking Pax7 (iPax7 -Dox and C2C12 myoblast and myotube controls) when Pax7-bound sites were examined exclusively (S4D Fig), whereas the comparisons of all sites exhibited cell-of-origin clustering (S4C Fig). Our analysis suggested that expression of this single factor could successfully reveal the impact of Pax7 binding on the open chromatin landscape and distinguish it from populations lacking this factor.

**Fig 4:**
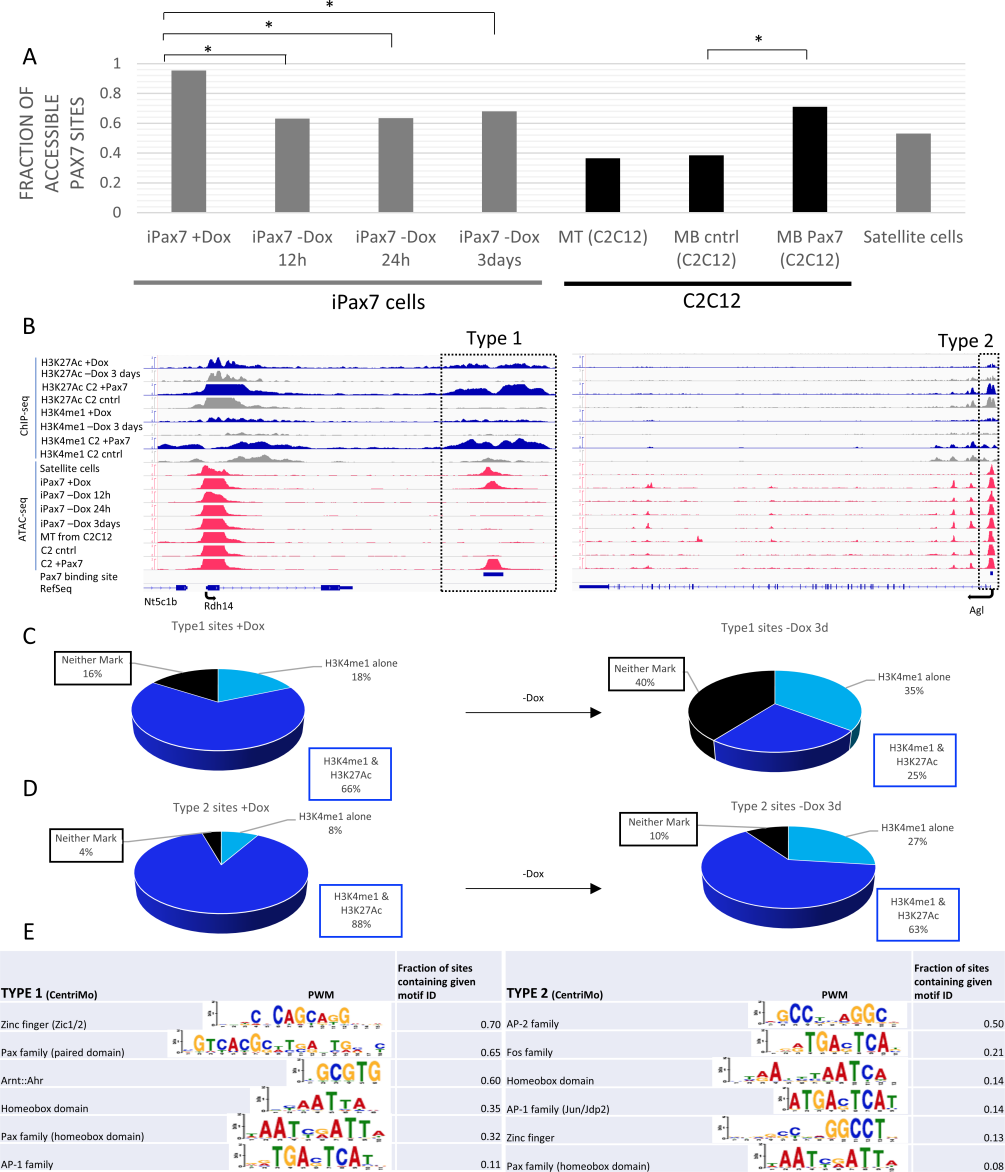
Pax7 functions to remodel chromatin. (**A**) Pax7 bound regions are accessible in the presence of Dox, and there is a significant and rapid loss of accessibility near Pax7 binding sites upon removal of Dox at 12h, 24h, and 3d (*, p<0.001). A significant number of sites bound by Pax7 in iPax7 cells also become accessible after ectopic expression of Pax7 in C2C12 cells. (**B**) Type 2, but not Type 1, sites (both denoted within dashed rectangles) remain accessible throughout myogenesis in satellite cells, iPax7 cells, myoblasts ectopically expressing Pax7, and myotubes and do not exhibit dramatic alterations in levels of H3K27Ac and H3K4me1 at these Pax7-bound regions. Type 1 sites further display *de novo* formation of open chromatin and enhancer marks in C2C12 myoblasts expressing Pax7. (**C**) Pie chart showing that the majority of Type 1 enhancers are lost upon removal of doxycyline whereas in **(D)** Type 2 enhancers do not display similar dramatic loss of enhancer modifications. MB and MT, myoblasts and myotubes, respectively. **(E)** Representative position-weight matrices (PWMs; adjusted p-value <0.05) found in CentriMo using MEME for a 250 bp window on both sides of the Pax7 peak center for Type 1 and Type 2 sites.

Next, we used *k*-means clustering to investigate chromatin accessibility and the epigenetic landscape as a function of Pax7 binding. We plotted the enrichment of accessible regions with respect to Pax7 binding sites, and these comparisons led to several notable observations (Fig 3). First, we found that nearly all (95%) Pax7 sites exhibited chromatin accessibility in the presence of Dox, suggesting that Pax7 could either enhance chromatin accessibility proximal to its binding sites or preferentially bind open chromatin (Figs 3 and 4A). Further, over half of these accessible Pax7 sites were also observed in satellite cells, and ~50% of these sites had not been previously identified as Pax7 targets in primary myoblasts [15], further indicating the importance of identifying Pax7 targets in myogenic precursors and bona fide satellite cells. Second, we identified at least two different types of Pax7-associated chromatin-accessible sites (Types 1 and 2): Type 1 sites exhibited a significant loss of accessibility immediately (within 12h) after Dox withdrawal, whereas the second group (Type 2) exhibited persistent accessibility in iPax7 cells up to 3d after Dox removal (Figs 3 and 4B). A sub-group showed persistent chromatin accessibility throughout this time course and maintained an accessible state in C2C12 myoblasts. These changes in accessibility were also mirrored by alterations in the enhancer signature, and likewise, the maintenance of accessibility was reflected by less drastic changes in enhancer histone modifications (Fig 4C and 4D; S5 Fig). Taken together with our studies of Pax7-dependent chromatin modifications, our results strongly suggest that we have identified functionally relevant Pax7 binding sites.

### Distinct motifs are associated with Type 1 and Type 2 sites

Members of the Pax gene family contain the highly conserved paired domain together with either a truncated or complete homeodomain. Pax7 contains the complete DNA-binding homeodomain, consistent with its known preference for homeobox-binding motifs in primary myoblasts [15]. We examined Type 1 and Type 2 sites separately using MEME to determine if the presence of distinct DNA sequence motifs can explain the differential accessibility for these two classes of Pax7 sites (Fig 4E). Overall, few motifs were shared between Type 1 and Type 2 sites, supporting the distinct fates of these potential enhancers after Pax7 removal. Interestingly, the Pax7 homeodomain and paired motifs were infrequently detected in Type 2 sites. Instead, the latter sites were highly enriched for AP-1/Jun/Fos family binding motifs together with the related AP-2 and AP-1/Jun dimerization protein 2 (Jdp2) binding sites. The enrichment of these motifs at Type 2 sites is consistent with the observed recruitment of c-Jun and Jdp2 at MyoD1 enhancers in myoblasts and myotubes (see below; [18]). On the other hand, we observed enrichment of the paired domain binding motif (shared by multiple Pax family proteins) and Pax7 homeobox domain binding sites in Type 1 sites. In addition, helix-loop-helix (Arnt/Ahr) and zinc finger family proteins, including Zic1, were predicted to bind Type 1 sites (Fig 4E). Of note, Pax3, which most closely resembles Pax7, was reported to interact with Zic1 [19], suggesting possible interplay between Pax family members and zinc-finger proteins at these sites in satellite cells. These results imply the existence of distinct types of Pax7 sites that are either rapidly remodeled to lose accessibility or that retain the hallmark of chromatin accessibility. We suggest that open chromatin associated with Type 1 sites strictly depends on the presence of Pax7, and therefore, Pax7 might open chromatin at these sites rather than bind to pre-existing open chromatin, whereas Type 2 sites could be “pre-opened” independent of Pax7 binding.

### Chromatin accessibility as an intrinsic property of myogenic precursors

**Fig 5:**
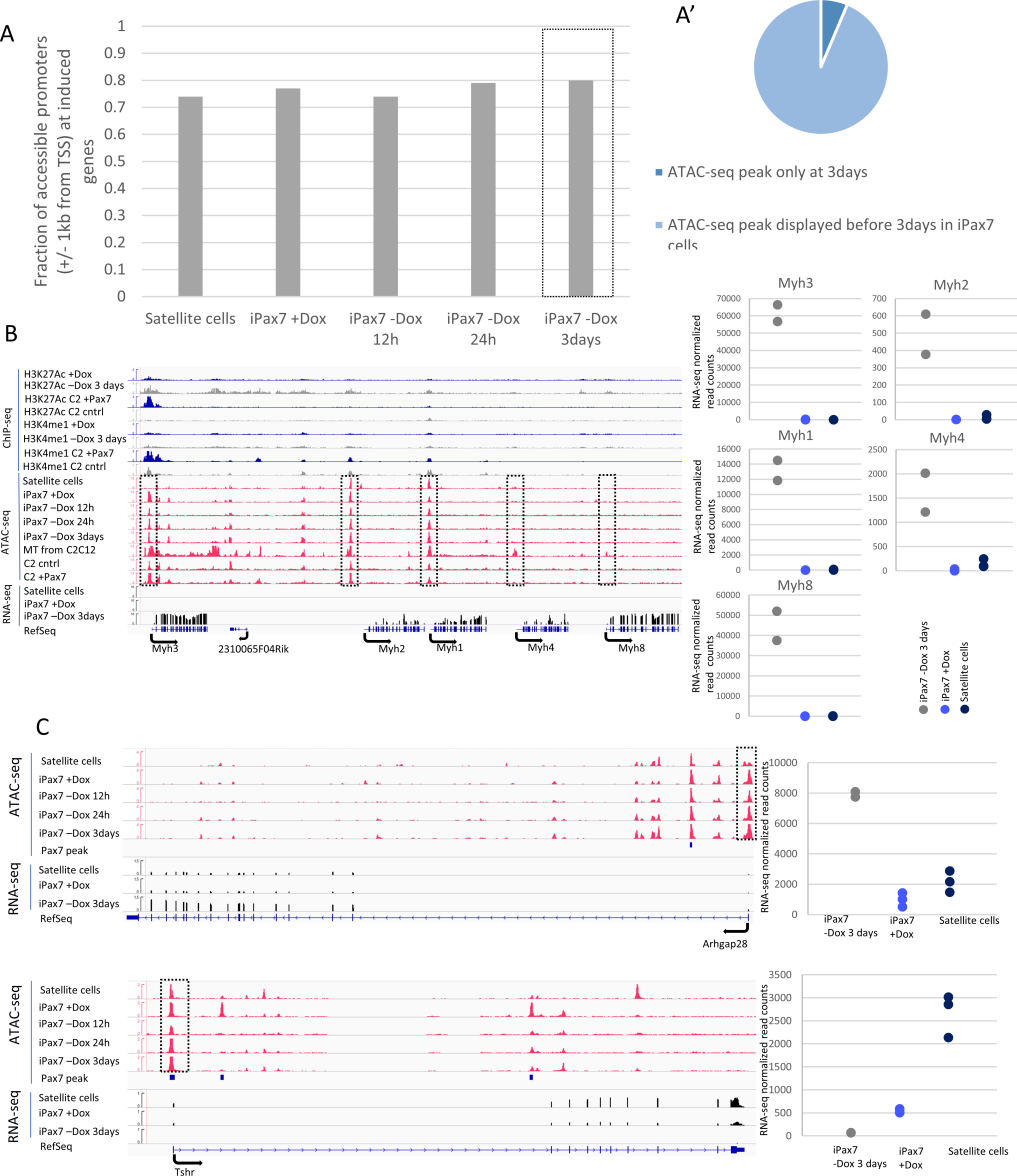
Chromatin accessibility in progenitors precedes differentiation-dependent gene expression. (**A**) The majority of Pax7 bound regions accessible at 3d post-Dox removal are already accessible in satellite cells, Dox-treated iPax7 cells, and earlier time points (Dox removal for 12h and 24h). (**A’**) Few sites (5.5%) exhibit *de novo* accessibility 3d after Dox removal. (**B**) Genes important for myogenesis and induced upon loss of Pax7 remain accessible throughout myogenesis, despite their relatively low expression. Normalized read densities for ATAC-seq and RNA-seq in iPax7 cells and satellite cells are indicated on the *y*-axis. Scatter plots of RNA-seq normalized read counts for replicates from satellite cells (n = 3), iPax7 +Dox (n = 3), and iPax7 -Dox after 3d (n = 2). (**C**) Accessibility at Pax7 sites throughout differentiation allows for gene expression plasticity. Genes that are up-regulated and down-regulated after loss of Pax7 retain accessibility at promoters throughout myogenesis. Read density for ATAC-seq and RNA-seq in iPax7 cells and satellite cells. Scatter plots showing normalized RNA-seq read counts for replicates from satellite cells, Dox-treated iPax7 cells, and iPax7 cells 3d after Dox removal. MB and MT, myoblasts and myotubes, respectively.

We also analyzed chromatin accessibility in iPax7 myogenic precursors globally, without reference to Pax7 binding, and restricted our analysis to promoters of all RefSeq genes. Since active genes generally show TSS-proximal chromatin accessibility, we investigated how chromatin accessibility changed genome-wide after Dox removal by examining accessible sites within 1 kb of all known TSSs in iPax7 cells with and without Dox, for a period up to three days, and in satellite cells (Fig 5). Surprisingly, we found that open chromatin, distinguished by strong ATAC-seq peaks, was readily apparent at promoters of genes that were not expressed in Dox-treated iPax7 cells or in satellite cells (Fig 5B). We focused on genes that were highly induced in cells differentiated to myotubes [14], including the *Myh* cluster, *Tnnc1*, and *Myog*. We confirmed both by RNA-seq and qRT-PCR that selected genes were indeed induced in iPax7 cells 3d after Dox removal, as they are in C2C12 myotubes (S6 Fig). Remarkably, our analysis suggested that nearly all accessible TSS-proximal sites discernible in a more differentiated state (3d after Dox removal) were already accessible in satellite cells or in iPax7 cells induced to express Pax7 (Fig 5B and 5C). These studies suggest that chromatin accessibility—a canonical feature of active gene expression—may already be defined in a precursor state (satellite cells and iPax7 cells expressing Pax7) and may signify an inherent property of plasticity within these myogenic precursors.

Intriguingly, other genes showed marked induction of expression 3d after Dox withdrawal and after myogenic differentiation in C2C12 cells without evidence of TSS-proximal chromatin accessibility (see *Myh4* and *Myh8*; Fig 5B). It is possible that other determinants of chromatin accessibility—beyond those that can be captured in our ATAC-seq experiments—confer inducibility on this group of genes.

### Pax7 binding alters chromatin in myoblasts

Our foregoing studies documented the effect of acutely removing Pax7, but the impact of expressing Pax7 in cells lacking this protein remained unanswered. It has been shown that iPax7 cells are able to reconstitute the satellite cell niche and to regenerate skeletal muscle in response to damage after transplantation [8, 9]. However, transplanted myoblasts are not able to support muscle regeneration in response to damage, suggesting that these cells have passed a lineage commitment barrier that circumscribes their ability to act as stem cells [20]. Therefore, a critical question is whether Pax7 binding leads to the modification of chromatin in cells that have already undergone the transition from satellite cells to myoblasts.

**Fig 6:**
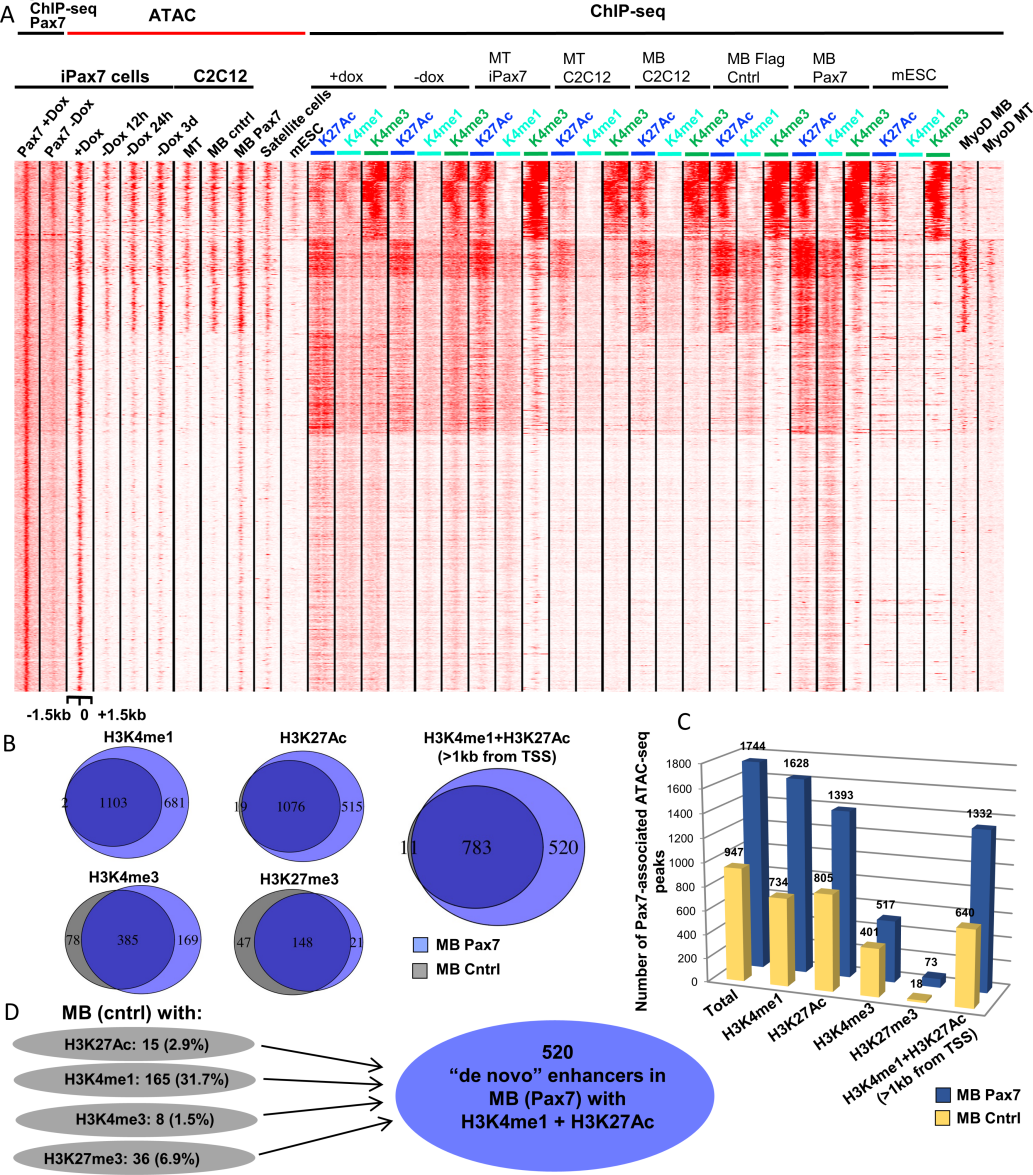
Induced accessibility of Pax7 binding sites associated with the formation of novel Pax7-specific enhancers. **(A)** mESC do not exhibit features of enhancers seen in myogenic precursors, and sites bound by MyoD in myoblasts are Pax7-specific as they lack chromatin accessibility in mESC. Histone modifications (H3K27Ac, H3K4me1, H3K4me3) and chromatin accessibility in myotubes (MT) differentiated from either iPax7 cells or C2C12 cells share similar patterns. MB from C2C12 with and without Flag control (Cntrl) also exhibit similar histone modification patterns. (**B**) Venn diagram depicting the number of shared and unique peaks with the indicated histone modifications at Pax7 binding sites (as defined in Dox-treated iPax7 cells) in control (MB Cntrl) and Pax7 expressing (MB Pax7) C2C12 cells. (**C**) Accessibility at Pax7 sites changes upon Pax7 expression in C2C12. Bar graph showing the quantification of accessible Pax7 binding sites with associated histone modifications at these sites in C2C12 control versus cells ectopically expressing Pax7. **(D)** Schematic showing the percentage of sites marked with individual histone modifications (as indicated) at known Pax7 binding sites in control C2C12 cells that undergo a novel conversion to an enhancer signature after Pax7 expression in C2C12 myoblasts.

To this end, we expressed Flag-tagged Pax7 in C2C12 myoblasts, which express a key determinant of skeletal muscle, MyoD1, and performed ChIP-seq to detect activating (H3K4me1/me3, H3K27ac) and repressive (H3K27me3) histone marks at genomic regulatory elements. We found that, as in iPax7 cells, the impact of Pax7 on H3K4me1 and H3K27ac was considerably more pronounced as compared to H3K4me3 or H3K27me3 (S7 Fig). We also performed ATAC-seq on C2C12 myoblasts expressing Flag-Pax7 and compared these data with a Flag-only control. As before, PCA of all ATAC-seq sites failed to distinguish between these populations, and vector-only and Flag-Pax7 expressing myoblasts clustered together (S4C Fig). However, when we considered only those accessible sites that overlapped with Pax7 bound regions, we were again able to cleanly separate both populations (S4D Fig). Interestingly, accessible sites in Flag-Pax7 expressing myoblasts more tightly clustered with satellite cells, suggesting that they acquired a state more highly related to the satellite cell population than vector-only controls. Consistent with this observation, *k*-means clustering showed stronger similarity in accessible sites when myoblasts expressing Pax7 were compared with satellite cells (Fig 6A; S7 Fig).

Importantly, these studies also showed that Pax7 expression was associated with pervasive opening of chromatin in C2C12 myoblasts at both Type 1 and Type 2 sites, although the most dramatic differences were observed at Type 1 sites, and the overlap with identifiable accessible sites in iPax7 cells was significant (Figs 3, 4 and 6). Indeed, we found that 74% of accessible, Pax7-bound sites identified in Dox-treated iPax7 cells were also captured after expression of Pax7 in C2C12 cells. Additionally, 51% of the Type 1 accessible sites—which were lost within 12h of Pax7 removal—were identified as newly accessible sites upon ectopic expression of Pax7 in C2C12 myoblasts, as compared to 3% of Type 1 sites found accessible in the Flag-only control. Moreover, we found that chromatin opening was accompanied by enhancement of H3K4me1 and H3K27ac, suggesting the potential activation of regulatory elements (Fig 6; S7 Fig). The most dramatic differences were again observed at sites identified as Type 1 (Figs 3 and 6). Interestingly, Pax7 appeared to robustly induce accessibility in regions that were previously inaccessible in C2C12 cells, and these sites strongly correlated with regions that lost accessibility after Pax7 removal in iPax7 cells. Importantly, accessibility of these Type 1 sites was not lost due to a general consequence of differentiation, as mESCs (from which iPax7 cells are derived) failed to display accessibility of these sites (Fig 6A; [21]). This observation further supports the notion that these sites are “opened” as a consequence of Pax7 expression. Inspection of these heatmaps and genomic regions (using the IGV browser and metagene analysis) indicated that nucleosomes in C2C12 or iPax7 cells assumed a “bimodal” configuration marked with H3K4me1 and H3K27ac in response to Pax7 binding (Figs 3 and 6; S7 Fig). These results reinforce our results with iPax7 cells before and after removal of Dox, since the presence of Pax7 was strongly associated with both of these active chromatin marks in a bimodal nucleosomal configuration.

These experiments also allowed us to explore the nature of chromatin marks at regions that are remodeled by Pax7, before and after expression of this regulatory protein. We found that Pax7 preferentially increased active chromatin marks (H3K27ac and H3K4me1) genome-wide, although there were less dramatic changes in the absolute number of regions marked with H3K4me3 or H3K27me3 (Fig 6B and 6C). Moreover, in iPax7 or C2C12 cells expressing Pax7, we found that H3K27 tri-methylated regions exhibited minimal overlap with Pax7 bound regions of chromatin in iPax7 cells cultured with Dox, irrespective of accessibility (Figs 3, 6B and 6C).

To further investigate the requirements for Pax7-dependent chromatin modifications, we examined the state of potential enhancers at distal regions (>1 kb from TSSs) able to bind Pax7 in muscle precursors. We examined regions marked by H3K27ac and H3K4me1 exclusively in C2C12 cells expressing Pax7 (but not in cells that do not express this factor) and calculated the extent to which these marks are reconfigured by this factor (Fig 6D). Interestingly, we found that a significant fraction of sites (~32%) was pre-marked with H3K4me1, but other modifications were either not present (H3K9me3) or were observed with very low frequency (H3K27ac, H3K27me3, and H3K4me3). These results suggest that (1) the majority of enhancers arise *de novo* after expression of Pax7, although a notable subset may be pre-marked by H3K4me1, and (2) Pax7 is largely unable to access and/or remodel chromatin that has assumed a silenced state.

Our findings lead to the following important conclusions. First, Pax7 is able to induce chromatin accessibility in lineage-committed myoblasts, and these sites recapitulate what is observed in myogenic precursors (iPax7 and satellite cells). In addition, Pax7 is primarily recruited to chromatin lacking repressive marks, H3K27me3 and H3K9me3.

We also explored chromatin accessibility and each of the active histone modifications in mouse ESC, since iPax7 cells are derived from this population. We aligned enriched ATAC-seq and ChIP-seq tags from ESC with the cognate Pax7-associated regions that we analyzed previously (Fig 6; [21, 22]). Strikingly, we found that few of these regions in ESC overlapped with accessible chromatin or displayed the H3K4me1^+^/H3K27ac^+^ enhancer signature apparent in satellite cells, Dox-treated iPax7 cells, and myoblasts expressing Pax7 (Fig 6A). Interestingly, the regions identified as accessible in ESC were highly enriched for H3K4me3, and indeed, this group was found to comprise the majority of promoters that were bound by Pax7, since 274 of 300 promoters (~91%) bound by this factor were located within this cluster. Most striking was the depletion of accessible sites and histone marks around Type 1 sites and Type 2 sites in ESC that subsequently recruit MyoD1 in skeletal muscle cells (see below). Our finding that accessible chromatin in ESC faithfully segregates with promoters suggests that a relatively small number of Pax7 sites are accessible in ESC and that they reside within promoters, yet the number of accessible regions vastly expands to include potential Pax7- and MyoD1-associated enhancers during the process of myogenic differentiation. Our data further suggest that Pax7 plays a fundamental role in recognizing and/or maintaining open chromatin and creating an active chromatin environment during the process of myogenic specification. Moreover, Pax7 also recognizes, opens up, and remodels accessible sites that were not previously accessible in myoblasts or mESC (Fig 6A), and accessibility could be maintained during myogenic differentiation on a subset of enhancers by another factor after Pax7 is no longer expressed (Type 2 sites). Since Pax7 can generate accessible sites de novo (Type 1), it could exhibit pioneer activity. When Pax7 is lost at these sites, nucleosome displacement is reversed, concomitant with the loss of a bimodal nucleosome signature.

### MyoD1 binding to regulatory elements rendered accessible by Pax7

Our finding that a subset of Pax7-bound regions retained an accessible chromatin signature with features of enhancers (typified by active histone marks and ATAC-seq peaks) subsequent to Dox removal prompted us to ask how this group might be distinguished from other genomic regions that *lose* these features. Myogenic enhancers are distinguished by the recruitment of several factors, including MyoD1 and c-Jun, together with H3K4me1 and H3K27ac deposition [18], and indeed, Type 2 Pax7 sites were found to be enriched for motifs associated with the AP-1/Jun/Fos family of proteins (Fig 4E). We further interrogated both groups of Pax7 bound regions by intersecting them with genomic regions bound by MyoD1 (ENCODE; [18, 22]). We also investigated Myogenin, an MRF that binds to the same E-box sequence as MyoD1 as cells differentiate to myotubes. We found that a notable fraction of these enhancers (26%) also bind MyoD1 in myoblasts and myotubes, and Myogenin was also recruited to these enhancers in differentiating myocytes (Fig 3A and 3B; S7 Fig). Most strikingly, we noticed that regions associated with Type 2 Pax7 sites were enriched for MyoD1 binding in myoblasts, whereas binding of this factor was less prevalent near Type I sites (Fig 3A). Further, we found that a significant fraction of these regions retained enhancer marks and indeed acquired a more characteristic enhancer appearance through the loss of H3K4me3. Sequence-specific transcription factors can cooperate as modules in skeletal muscle enhancers [18]. Therefore, we investigated the spatial characteristics associated with MyoD1 and Pax7 binding. Interestingly, we found that binding sites for these factors were indeed close to one another: the median distance between the two sites was 337 bp and 236 bp for promoters and enhancers, respectively (Fig 3B).

Taken together, our studies suggest several key conclusions. First, Pax7 binding sites are non-equivalent and can be segregated into at least two different types depending upon whether they lose their accessibility and whether or not they recruit MyoD1, and possibly other factors (Fig 4E), to proximal sites. Second, Pax7 could serve a pioneering role in which MRFs, such as MyoD1 and Myog, subsequently bind to enhancers that recruited Pax7 at an earlier stage in muscle development (Fig 6; S7 Fig). Therefore, it is possible that MyoD1 maintains chromatin “openness” and, potentially, enhancer activity, during differentiation, when Pax7 is no longer expressed.

Beyond their potential for therapeutic applications, our studies suggest the utility of iPax7 cells for the dissection of epigenetic mechanisms associated with skeletal muscle precursors and satellite cells. The ability to rapidly shut off Pax7 expression will provide a powerful tool, enabling an analysis of the kinetics of chromatin remodeling at target genes, an interesting question in light of our findings that suggest acute alterations at certain genomic regions within 12h of Pax7 disappearance. Further, this system allowed us to study this factor in myogenic precursors that express Pax7 at levels that more closely emulate those of SC. By comparing features of chromatin in our myogenic precursors and satellite cells, we also showed that Pax7 functions to remodel the adjacent landscape, and based on its ability to open chromatin at enhancers in both populations, we conclude that we have identified authentic Pax7 targets. Our results therefore vastly extend previous studies in myoblasts aimed at Pax7 target identification [15]. Indeed, we have identified a set of targets that are likely to exist only in precursors but not in myoblasts: such targets may no longer be accessible to Pax7 binding after differentiation to myoblasts.

Our results invite mechanistic comparisons with pituitary differentiation, in which the binding of Pax7 could prefigure the subsequent recruitment of the T-box transcription factor, Tpit, thus implicating Pax7 as a pioneer factor that facilitates an open and active chromatin state [23]. In this regard, we have found that Pax7 is able to open chromatin in muscle cells, but it does so primarily at sites that are depleted of repressive histone modifications, such as H3K27me3 and H3K9me3, which are associated with condensed or heterochromatic regions (Fig 6D). Further, at one group of loci (Type 1), Pax7 is required to maintain open chromatin, yet Pax7 is not required to maintain open chromatin at other (Type 2) sites. We have also found that Pax7 sites reside a short distance from future sites bound by MyoD1 after lineage determination. Since MyoD1 is highly enriched at enhancers [18], this MRF is a likely candidate to maintain an active chromatin state after Pax7 expression is extinguished, and in this sense, the Pax7-MyoD1 relationship could parallel that of Pax7 and Tpit in the pituitary. MyoD1 could thus constitute a “settler” transcription factor that takes advantage of an open chromatin environment afforded by Pax7 [24]. Future studies will be required to identify other factors that could promote Pax7 recruitment to chromatin and subsequently sustain the features of open chromatin instigated by Pax7.

Our results also lead to several mechanistic predictions regarding Pax7 function that will require future study. For example, our work suggests that Pax7 may play a more pronounced role at enhancers through the recruitment of co-factors and chromatin modifying enzymes that specifically alter the balance of H3K4 mono- versus tri-methylation, as well as H3K27 acetylation. Distinct MLL family members could preferentially impact promoters and enhancers, since MLL3/MLL4 complexes have been shown to mono-methylate H3K4 and drive H3K27 acetylation at enhancers, whereas MLL1/MLL2 could play a larger role in generating H3K4 tri-methylation at promoters [25–29]. Therefore, it will be important to examine the role of each complex in Pax7-mediated chromatin remodeling and enhancer assembly. *In vitro* binding and co-immunoprecipitation experiments suggest that MLL1 and MLL2 can interact with Pax7 [30, 31], and other studies suggest that an adaptor protein (Pax3/7BP) can bridge Pax3 and Pax7 with each of the four MLL proteins [32]. We also found that Pax7 was, in general, unable to remodel facultative heterochromatin, suggesting that recruitment of Pax7 is likely to require additional features beyond its DNA sequence motifs.

Transplantation of *in vitro* expanded satellite cells or myoblasts is unable to reconstitute the satellite cell niche or robustly support muscle regeneration [8, 33, 34], suggesting that they have lost the requisite plasticity needed to reconstitute the stem cell compartment. We have shown that Pax7 is responsible for remodeling chromatin and inducing epigenetic characteristics associated with satellite cells, but it is likely that other transcription factors in addition to Pax7 will be required to fully reprogram myoblasts toward a precursor state. By providing a valuable resource for those interested in epigenetic regulation of muscle stem cell identity, our experiments using these precursors set the stage (1) to begin identifying these factors, (2) to understand the basis of plasticity, exemplified by our finding that chromatin accessibility of promoters precedes gene activation, and (3) to understand how epigenetic marks are remodeled in aging satellite cells.

## Materials and methods

### Cell culture

iPax7 cells were generated as described (Darabi et al., 2011). iPax7 cells were grown on 0.1% gelatin-coated culture plates in GlutaMAX supplemented IMDM (Gibco) with 15% stem cell qualified fetal bovine serum (Gemini), 200 μg/ml bovine holo-transferrin (Sigma), 10 μg/ml ascorbic acid, 4.5 mM 1-thioglycerol, 100 IU penicillin, and 100 μg/ml streptomycin. Media was changed every two days with the addition of 5 ng/ml recombinant mouse FGF basic protein (R&D systems), and 0.75 μg/ml doxycycline (Dox) for the expression of Pax7. As indicated, iPax7 cells grown in -Dox condition were washed with PBS and switched to media lacking Dox. C2C12 myoblasts (Sigma) were grown in DMEM (cat #15-013-CV Corning) supplemented with 100 IU penicillin, 100 μg/ml streptomycin, 2 mM L-Glutamine and 10% fetal bovine serum, and differentiated to myotubes by incubating cells with DMEM supplemented with 2% horse serum. Stable cell lines ectopically expressing carboxy-terminal 3xFlag-tagged Pax7 or 3xFlag-only control were generated as described [35]. Pax7 cDNA was sub-cloned from p2lox Pax7 into pBABE-puro (Addgene) and constructs were transfected into Phoenix-Eco cells. Harvested viral supernatant was used to transduce C2C12 myoblasts together with polybrene and selected with puromycin (4 μg/ml).

### ChIP-seq and metagene analysis

ChIP-seq libraries (GSE89977) were prepared as described [14] and sequenced (51 bp, single-end reads) using an Illumina HiSeq2500 machine. Raw reads were aligned to the *Mus musculus* reference genome (mm9 assembly), and non-duplicate, uniquely mapped reads were selected for downstream processing as previously described [25, 35]. Peak-calling was performed using Qeseq [36] with a default p-value of 0.05. For each histone mark, duplicate reads were removed, replicates were merged, and peaks were called from merged data. For Pax7 ChIP-seq, each replicate was used to generate peaks for each condition (+Dox and -Dox). The final list of Pax7-bound sites was generated by retaining peaks that were (1) found in two or more +Dox-treated samples and (2) removing peaks that were found in any replicate without Dox treatment. For data visualization, (1) each read was extended in the 3' direction by the average DNA fragment length, (2) the number of reads at each locus was normalized per million total reads for each respective experiment, and (3) the resulting data were visualized in Integrative Genomics Viewer (IGV)[37, 38]. Fragment lengths were 150 bp (histone marks in iPax7 +Dox and iPax7 -Dox cells), 200 bp (myotubes differentiated from iPax7 cells), 230 bp (C2C12 myoblasts over-expressing Flag control or Pax7-Flag protein), and 250 bp (Pax7 ChIP in iPax7 cells). Previously published ChIP-seq data were processed as above. These include TAP-tagged Pax7 ChIP in primary myoblasts (GSE25064;[15]); MyoD1 and Myogenin ChIP in C2C12 myoblasts and myocyte differentiated C2C12 myoblasts (GSE36024, Mouse ENCODE, Wold Laboratory, Marinov Laboratory, Trout Laboratory [22]); H3K4me3 and H3K27me3 in ASC (GSE47362; (Liu et al., 2013)); and H3K4me1, H3K4me3, H3K27me3, and H3K27ac ChIP in mouse Bruce4 ESC (GSE31039, Mouse ENCODE, Ren Laboratory, Shen Laboratory [22]).

For metagene analyses, profile plots of average ChIP-seq signals were made using the computeMatrix tool of deepTools version 2.2.3 (Ramirez, Ryan et al. 2016). Using computeMatrix reference-point, scores for each bigwig file used in the plot were calculated around genomic regions defined as a 1.5 kb window upstream and downstream of the peak centers of Pax7 binding sites. In analyses examining TSS regions, RefSeq TSS regions were used as a reference-point. Output from the computeMatrix tool was subsequently used to generate profile plots using the plotProfile tool of deepTools.

### ATAC-seq

Cells were harvested by trypsinization and resuspended in appropriate media. 500,000 cells were subsequently used for nuclear extraction, except for satellite cells (described separately) and myotubes where 100,000 cells were used to adjust for their multinucleated nature. Cells were washed with 500 μl cold PBS and resuspended in 500 μl cold lysis buffer (10mM Tris-HCl pH 7.4, 10mM NaCl, 3mM MgCl_2_, 0.1% IGEPAL CA-630), and spun at 500g for 10 minutes at 4°C. Supernatant was discarded and the nuclear pellet was resuspended in nuclease-free water and 50,000 cells (10,000 cells for myotubes) in 22.5 μl nuclease free water was mixed with 25 μl TD Buffer and 2.5 μl Tn5 Transposase (Illumina cat #FC-121-130). 50,000 freshly sorted satellite cells from *Pax7*^*ZsGreen*^ mice were washed with 200 μl of cold PBS then resuspended in 100 μl of cold lysis buffer, spun at 500 g for 10 minutes at 4°C and resuspended in 50 μl of the transposition reaction mix. Transposition occurred at 37°C for 30 minutes, after which transposed DNA was purified using a Qiagen MinElute Kit and eluted in 10 μl Elution Buffer. Transposed DNA fragments were mixed with 7 μl nuclease free H_2_O, 2.5 μl of each forward and reverse 25 μM stock of Nextera PCR primers, 3 μl of 1000x diluted stock of SYBR Green I (Invitrogen #S-7563), 25 μl NEBNext High-Fidelity 2x PCR Master Mix (New England BioLab #M0541). All samples were amplified for 5 cycles (as described in [17]) after which 5 μl was aliquoted to determine optimal cycle numbers. 5 μl was mixed with 1.9 μl Nuclease Free H_2_O, 1.25 μl of each forward and reverse customized 5 μM stock of Nextera PCR primers, 0.6 μl of 1000x diluted stock of SYBR Green I, and 5 μl NEBNext High-Fidelity 2x PCR Master Mix. All samples were amplified for 20 cycles, and the number of additional cycles to the remaining transposed DNA was determined as described by [17]. Libraries were then purified with 1.2x vol AMPure (Beckman) beads. Tagmentation was confirmed via Tapestation.

ATAC-seq libraries (GSE89977) were sequenced (51 bp, paired end) on an Illumina HiSeq2500 machine. Raw reads were aligned to the *Mus musculus* mm9 reference genome with Bowtie2 version 2.2.6 using the options ‘—local—dovetail—minins 38—maxins 2000—no-mixed—no-discordant’. PCR and optical duplicates were removed with Picard. Prior to downstream analysis, mitochondrial reads were removed and the coordinates of the remaining reads were corrected to account for the 9 bp insert introduced by the Tn5 transposase by offsetting the 5' ends by either +4 (for plus strand reads) or -5 (for minus strand reads). Replicate experiments were merged and reads were normalized per million total reads (as above) for data visualization in IGV. Transposase-accessible regions were identified using the callpeak command of MACS2 version 2.0.10 with options ‘—nomodel—nolambda—keep-dup all—call-summits—f BAM—g mm’ as described [39]. Statistical tests for altered accessibility of Pax7 sites upon loss or gain of Pax7 expression were performed using a Chi-square test. mESC ATAC-seq data were obtained from GEO using the following entry: ATAC-Seq 50k mESC (GSM2156965) [21].

## Supporting information

S1 Fig Selection and partial characterization of iPax7 myogenic precursors. (**A**) iPax7 cells lose nuclear Pax7 and assume a more differentiated identity concomitant with morphological changes upon removal of Pax7 (-Dox), as shown by phase-contrast (A) and immunofluorescence images (A’). (**B**) iPax7 cells commit to a myogenic program upon loss of Pax7, and satellite cell marker Pax7 is rapidly lost upon removal of Dox (-Dox, 12h). Western blot analysis of proteins indicated at right. Loss of Pax7 in iPax7 cells is accompanied by decreased expression of Myf5, whereas Myog and Myosin Heavy Chain (MHC, a terminal differentiation marker) levels increased. Tubulin, loading control. (**C**) Immunoflourescence staining illustrating the myogenic differentiation potential of iPax7 cells in the absence of Dox at 3d and during incubation with horse serum (HS) for 3d. iPax7 cells in -Dox 3d and HS 3d conditions exhibited reduced nuclear staining of the early MRF Myf5 concomitant with increased nuclear staining of the late MRF Myog as well as cytoplasmic MHC.
(TIF)

S2 Fig RNA-seq analysis of satellite cells and iPax7 cells with and without Dox treatment. (**A**) Pearson correlation plot showing Pax7 expression in iPax7-cell +Dox promotes a state more similar to satellite cells than iPax7 cells without Dox. (**B**) Gene ontology categories enriched for genes up-regulated upon loss of Pax7 that are also expressed in satellite cells (green, left). Gene ontology categories enriched for genes down-regulated upon loss of Pax7 that are expressed in satellite cells (red, right) are also indicated. **(C)** Comparisons of H3K4me3 and H3K27me3 at promoter regions in activated satellite cells (ASC; (Liu et al., 2013)), Dox-treated iPax7 cells, and C2C12 myoblasts (MB) and myotubes (MT). Scatter plots show ChIP-seq tag densities (in reads per million, RPM) for each mark.
(TIF)

S3 Fig Validation of selected Pax7 targets. (**A**) Confirmation of selected Pax7 targets using ChIP and qPCR in +Dox versus -Dox conditions. (**B**) 50% of the Pax7 targets identified by ChIP-seq in iPax7 cells are found in a previous study that employed over-expression of tagged Pax7 in primary myoblasts (Soleimani et al., 2012). (B’) Homeobox domain and paired domain motifs were found in Pax7 binding sites. MEME search was restricted to a 250 bp window on both sides of the peaks of Pax7 enrichment. (**C**) Gene ontology categories associated with genes whose TSS is closest to the Pax7 binding sites.
(TIF)

S4 Fig Principal component analysis (PCA) of Pax7-dependent chromatin accessibility. (**A**) PCA plot indicates that ATAC-seq accessible sites cluster according to cell-of-origin. iPax7 cell samples: iPax7 +Dox (n = 4), iPax7 -Dox 12h (n = 3), iPax7 -Dox 24h (n = 3), iPax7 -Dox 3 days (n = 4). C2C12 samples: Myotubes (MT) (n = 3), Myoblasts (MB) with Flag control (n = 3), Myoblasts with Pax7-flag (n = 4). (**B**) ATAC-seq data in panel A were re-analyzed, restricting the analysis to Pax7 bound regions only. (**C**) PCA-plot for all ATAC-seq accessible sites for all replicates included in panel A. Populations again cluster according to cell-of-origin with the addition of satellite cells. (**D**) PCA plot for all samples included in panel C, but data were restricted to Pax7 binding sites. Pax7 expression generates ATAC-seq profiles that are distinct from conditions without induced Pax7 expression and that more closely resemble satellite cells at Pax7 binding sites. Red, dashed rectangles indicate how populations re-cluster upon restricting the analysis to Pax7-enriched sites.
(TIF)

S5 Fig The epigenetic landscape associated with iPax7 cells. IGV browser snapshots of ChIP-seq, ATAC-seq, and RNA-seq data are shown. Normalized read densities are indicated on the *y*-axis.
(TIF)

S6 Fig Expression analysis of myogenic markers upon spontaneous differentiation of iPax7 cells. qRT-PCR of iPax7 cells cultured with (+Dox) or without (-Dox) for 12h, 24h, or 3d. Fold-enrichment (*y*-axis) was plotted relative to Dox-treated iPax7 cells, whose values were set to 1. For reference, expression was also compared with C2C12 myoblasts (MB) and myotubes (MT).
(TIF)

S7 Fig Pax7 bound enhancers are specific to myogenic lineages. (**A**) Heatmap displaying acquisition of enhancer marks and increased chromatin accessibility (at Type 1 sites) after Pax7 expression in C2C12 myoblasts. ATAC-seq and ChIP-seq data are shown for iPax7 cells with or without Dox (3d) and for C2C12 with (MB Pax7) or without (MB Cntrl) Pax7 expression. Pax7 bound regions gaining an enhancer signature in C2C12 cells are not universally pre-bound by MyoD nor Myog. MB and MT, myoblasts and myotubes, respectively. **(B)** Metagene analysis showing the average ChIP-seq signal for Pax7 (iPax7 +Dox vs -Dox) and indicated epigenetic marks (H3K27Ac, H3K4me1, H3K4me3, H3K27me3) in C2C12 myoblasts over-expressing Pax7 (MB-Pax7) or Flag-only control (MB-cntrl) C2C12 myoblasts plotted for regions 1.5 kb upstream and downstream of the center of Pax7 binding sites. Levels of enhancer marks and bimodality increased upon introducing Pax7 in C2C12 myoblasts.
(TIF)

S1 Table RNA-seq. RNA-seq expression table for iPax7 –Dox 3d, iPax7 cells +Dox and Satellite cells. Associated values are sample-averages of normalized read-counts for genes expressed in Satellite cells and iPax7 cells in +Dox condition.
(XLSX)

S2 Table Pax7 binding sites. BED-format of Pax7 binding sites (chromosome name, starting position and end position) and the gene name of its most proximal transcriptional start site calculated from the center of each binding site.
(XLSX)

S1 Text Supplemental materials and methods. (PDF)
