# Supplementary figures and images for: Pax7 remodels the chromatin landscape in skeletal muscle stem cells
Source: PLoS One. 2017 Apr 25;12(4):e0176190. doi: 10.1371/journal.pone.0176190 (PMC5404880; doi:10.1371/journal.pone.0176190)

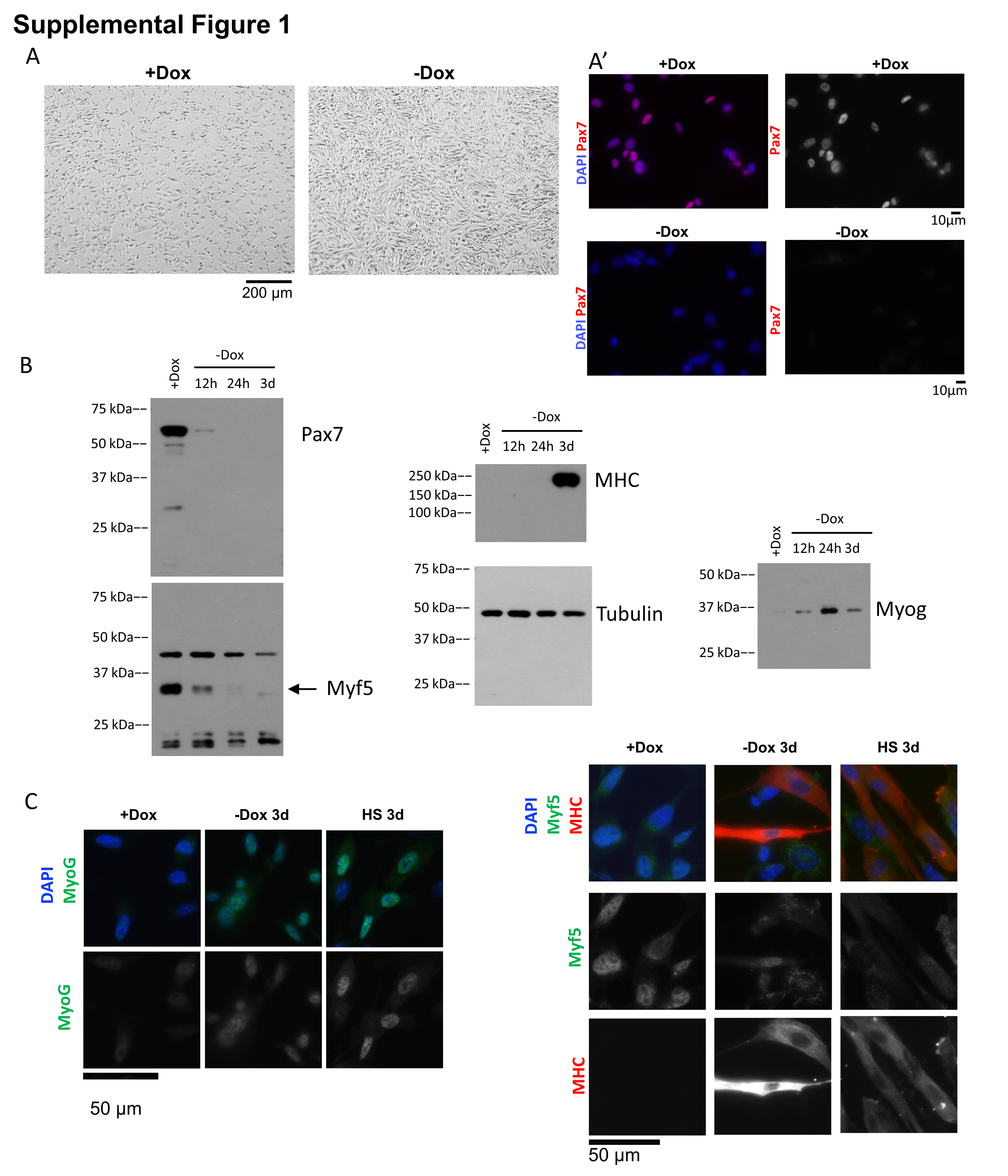

Supplement: S1 Fig — (A) iPax7 cells lose nuclear Pax7 and assume a more differentiated identity concomitant with morphological changes upon removal of Pax7 (-Dox), as shown by phase-contrast (A) and immunofluorescence images (A’). (B) iPax7 cells commit to a myogenic program upon loss of Pax7, and satellite cell marker Pax7 is rapidly lost upon removal of Dox (-Dox, 12h). Western blot analysis of proteins indicated at right. Loss of Pax7 in iPax7 cells is accompanied by decreased expression of Myf5, whereas Myog and Myosin Heavy Chain (MHC, a terminal differentiation marker) levels increased. Tubulin, loading control. (C) Immunoflourescence staining illustrating the myogenic differentiation potential of iPax7 cells in the absence of Dox at 3d and during incubation with horse serum (HS) for 3d. iPax7 cells in -Dox 3d and HS 3d conditions exhibited reduced nuclear staining of the early MRF Myf5 concomitant with increased nuclear staining of the late MRF Myog as well as cytoplasmic MHC. (TIF) [file pone.0176190.s001.tif]

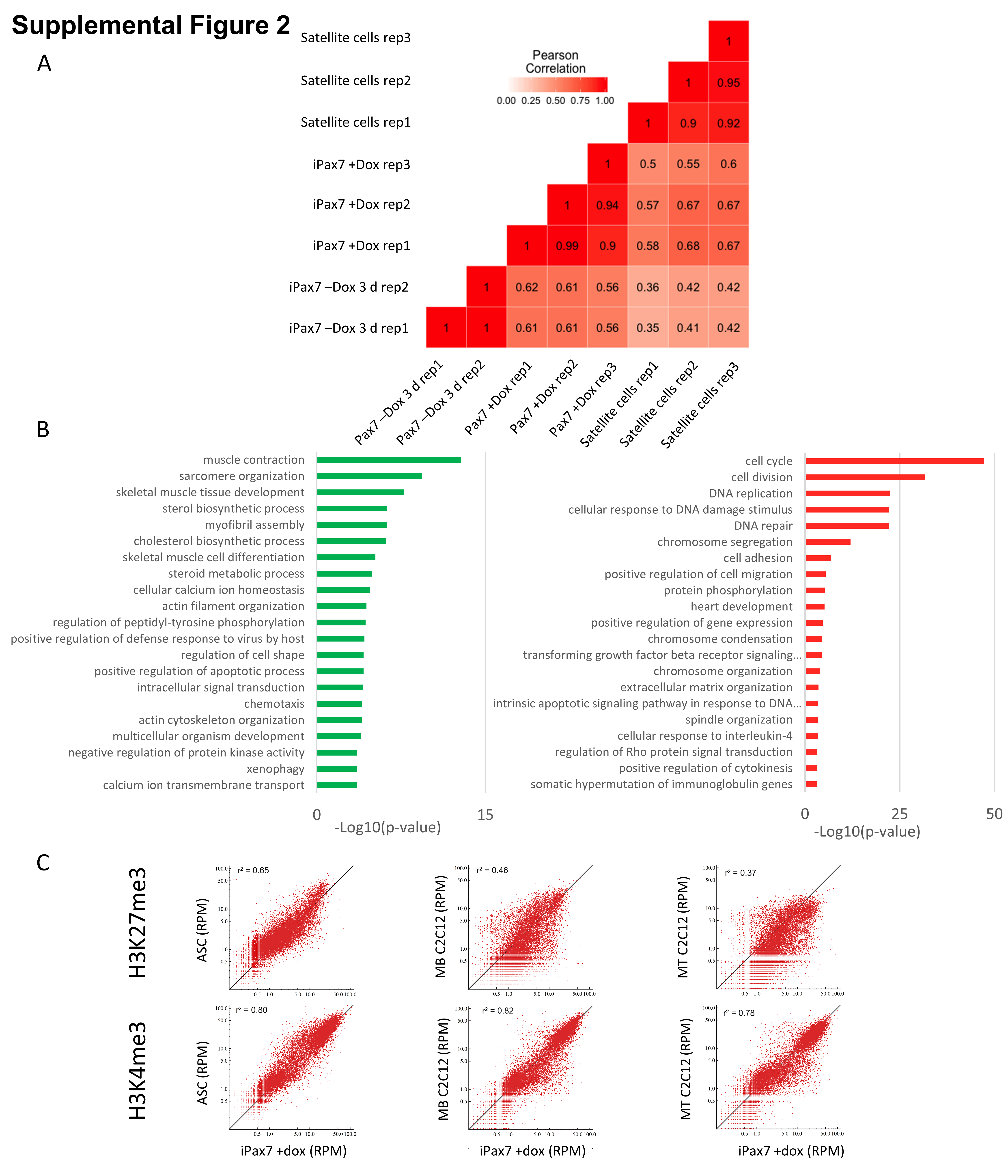

Supplement: S2 Fig — (A) Pearson correlation plot showing Pax7 expression in iPax7-cell +Dox promotes a state more similar to satellite cells than iPax7 cells without Dox. (B) Gene ontology categories enriched for genes up-regulated upon loss of Pax7 that are also expressed in satellite cells (green, left). Gene ontology categories enriched for genes down-regulated upon loss of Pax7 that are expressed in satellite cells (red, right) are also indicated. (C) Comparisons of H3K4me3 and H3K27me3 at promoter regions in activated satellite cells (ASC; (Liu et al., 2013)), Dox-treated iPax7 cells, and C2C12 myoblasts (MB) and myotubes (MT). Scatter plots show ChIP-seq tag densities (in reads per million, RPM) for each mark. (TIF) [file pone.0176190.s002.TIF]

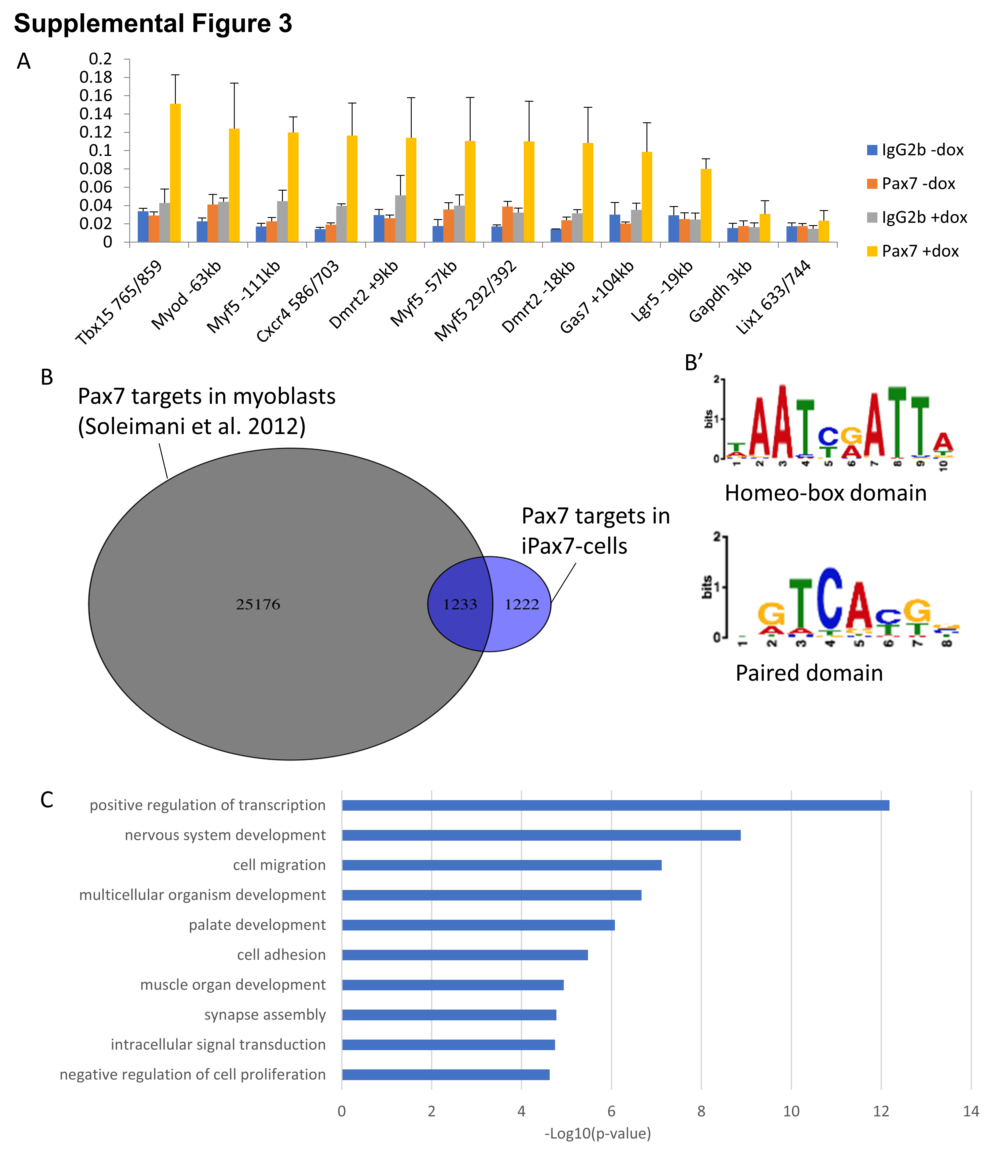

Supplement: S3 Fig — (A) Confirmation of selected Pax7 targets using ChIP and qPCR in +Dox versus -Dox conditions. (B) 50% of the Pax7 targets identified by ChIP-seq in iPax7 cells are found in a previous study that employed over-expression of tagged Pax7 in primary myoblasts (Soleimani et al., 2012). (B’) Homeobox domain and paired domain motifs were found in Pax7 binding sites. MEME search was restricted to a 250 bp window on both sides of the peaks of Pax7 enrichment. (C) Gene ontology categories associated with genes whose TSS is closest to the Pax7 binding sites. (TIF) [file pone.0176190.s003.TIF]

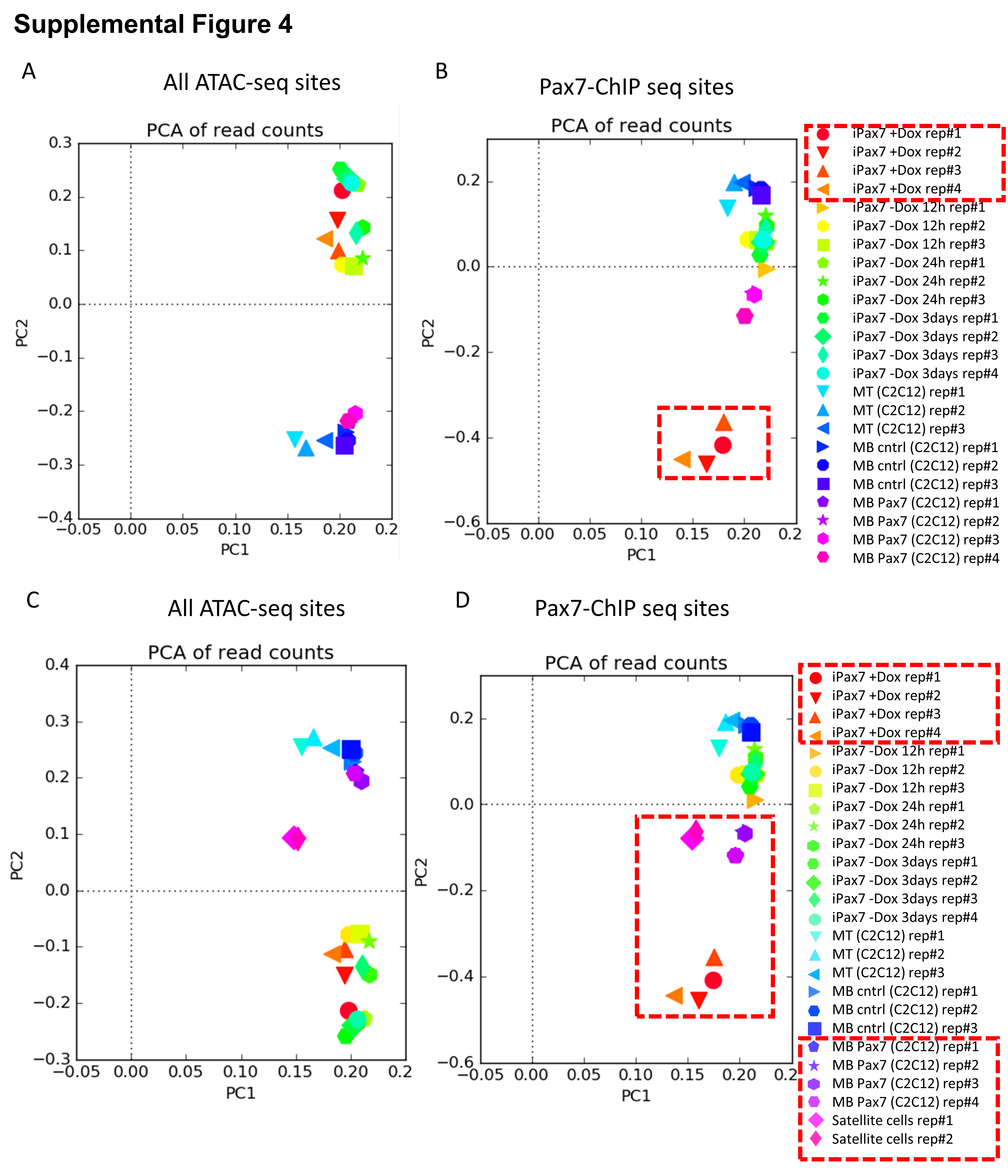

Supplement: S4 Fig — (A) PCA plot indicates that ATAC-seq accessible sites cluster according to cell-of-origin. iPax7 cell samples: iPax7 +Dox (n = 4), iPax7 -Dox 12h (n = 3), iPax7 -Dox 24h (n = 3), iPax7 -Dox 3 days (n = 4). C2C12 samples: Myotubes (MT) (n = 3), Myoblasts (MB) with Flag control (n = 3), Myoblasts with Pax7-flag (n = 4). (B) ATAC-seq data in panel A were re-analyzed, restricting the analysis to Pax7 bound regions only. (C) PCA-plot for all ATAC-seq accessible sites for all replicates included in panel A. Populations again cluster according to cell-of-origin with the addition of satellite cells. (D) PCA plot for all samples included in panel C, but data were restricted to Pax7 binding sites. Pax7 expression generates ATAC-seq profiles that are distinct from conditions without induced Pax7 expression and that more closely resemble satellite cells at Pax7 binding sites. Red, dashed rectangles indicate how populations re-cluster upon restricting the analysis to Pax7-enriched sites. (TIF) [file pone.0176190.s004.TIF]

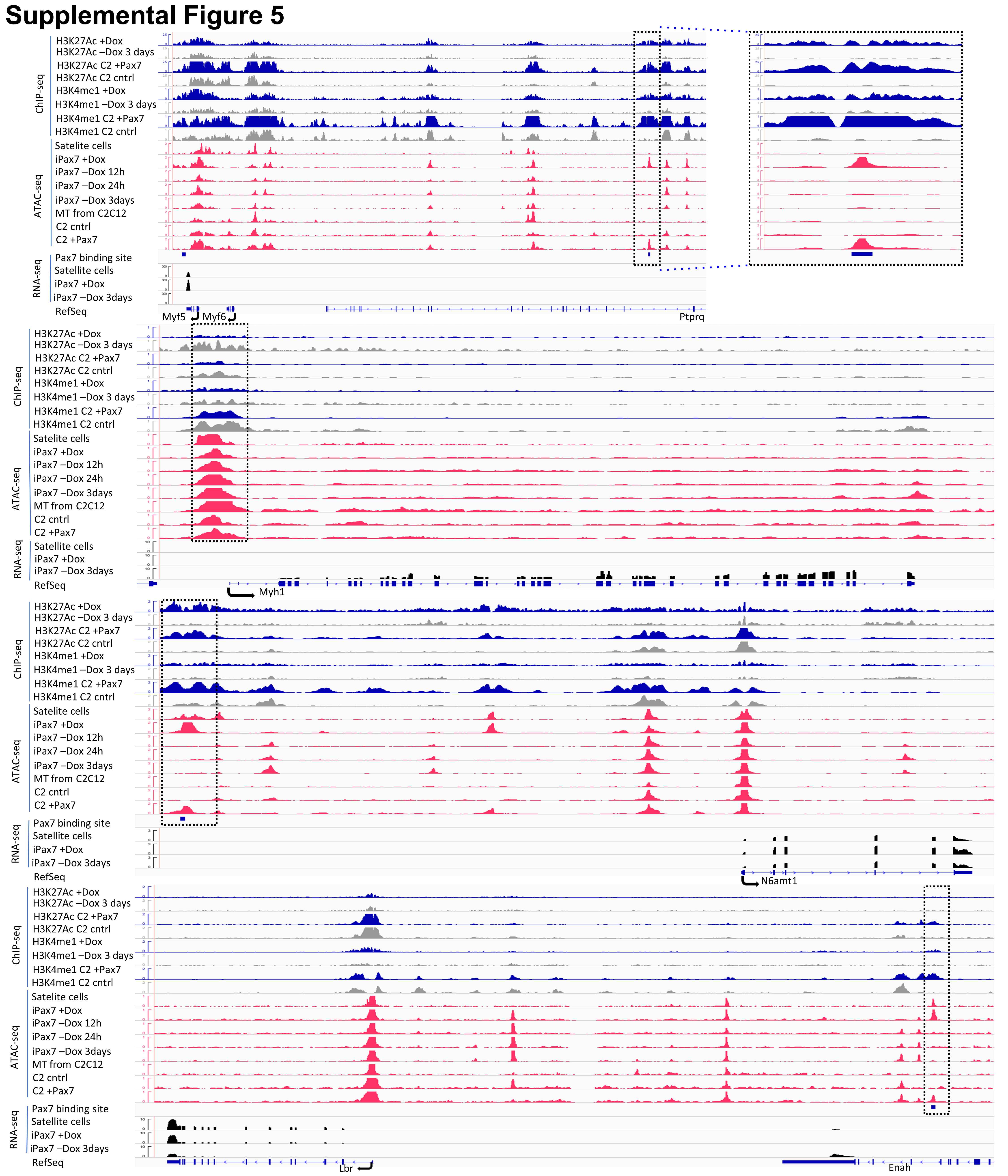

Supplement: S5 Fig — IGV browser snapshots of ChIP-seq, ATAC-seq, and RNA-seq data are shown. Normalized read densities are indicated on the y-axis. (TIF) [file pone.0176190.s005.TIF]

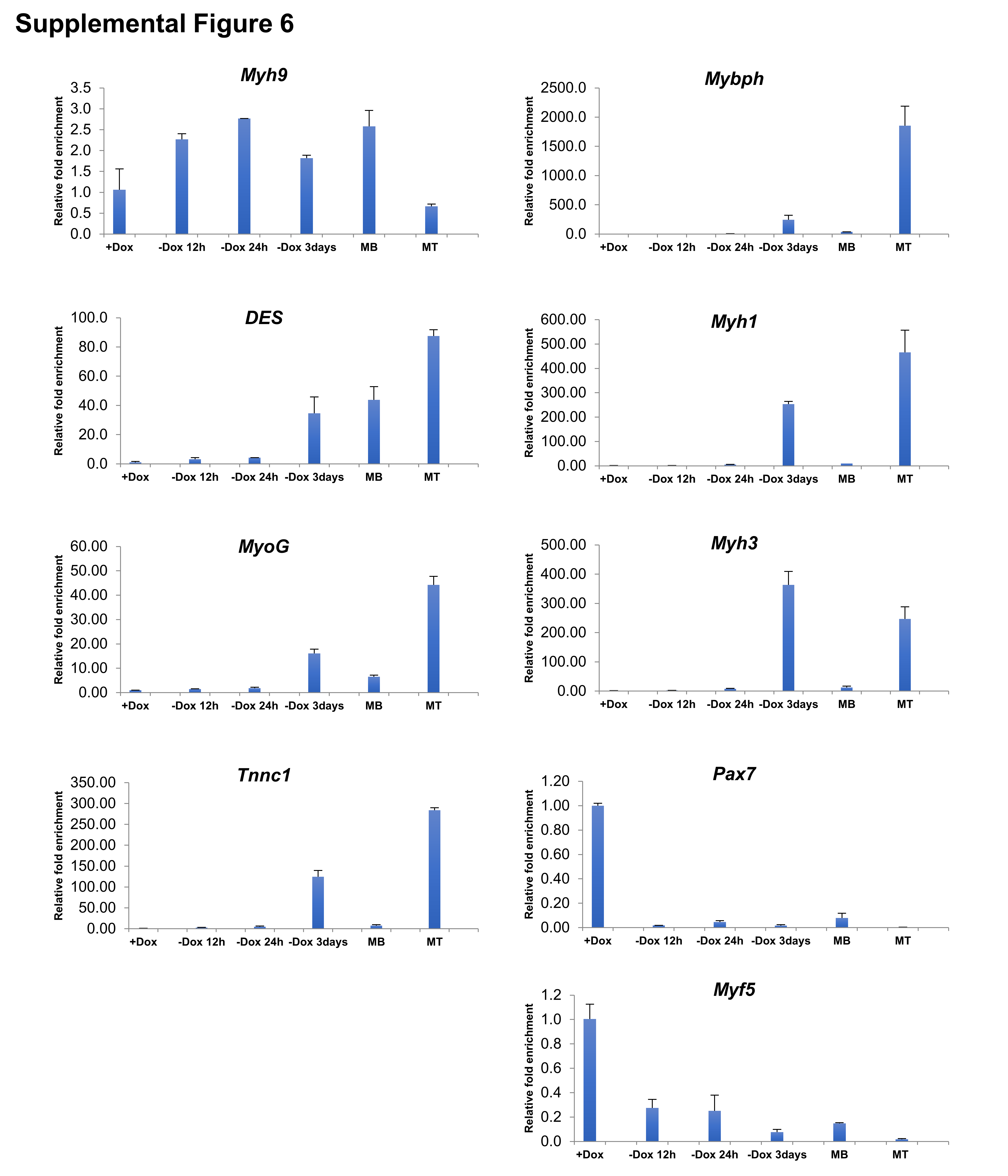

Supplement: S6 Fig — qRT-PCR of iPax7 cells cultured with (+Dox) or without (-Dox) for 12h, 24h, or 3d. Fold-enrichment (y-axis) was plotted relative to Dox-treated iPax7 cells, whose values were set to 1. For reference, expression was also compared with C2C12 myoblasts (MB) and myotubes (MT). (TIF) [file pone.0176190.s006.TIF]

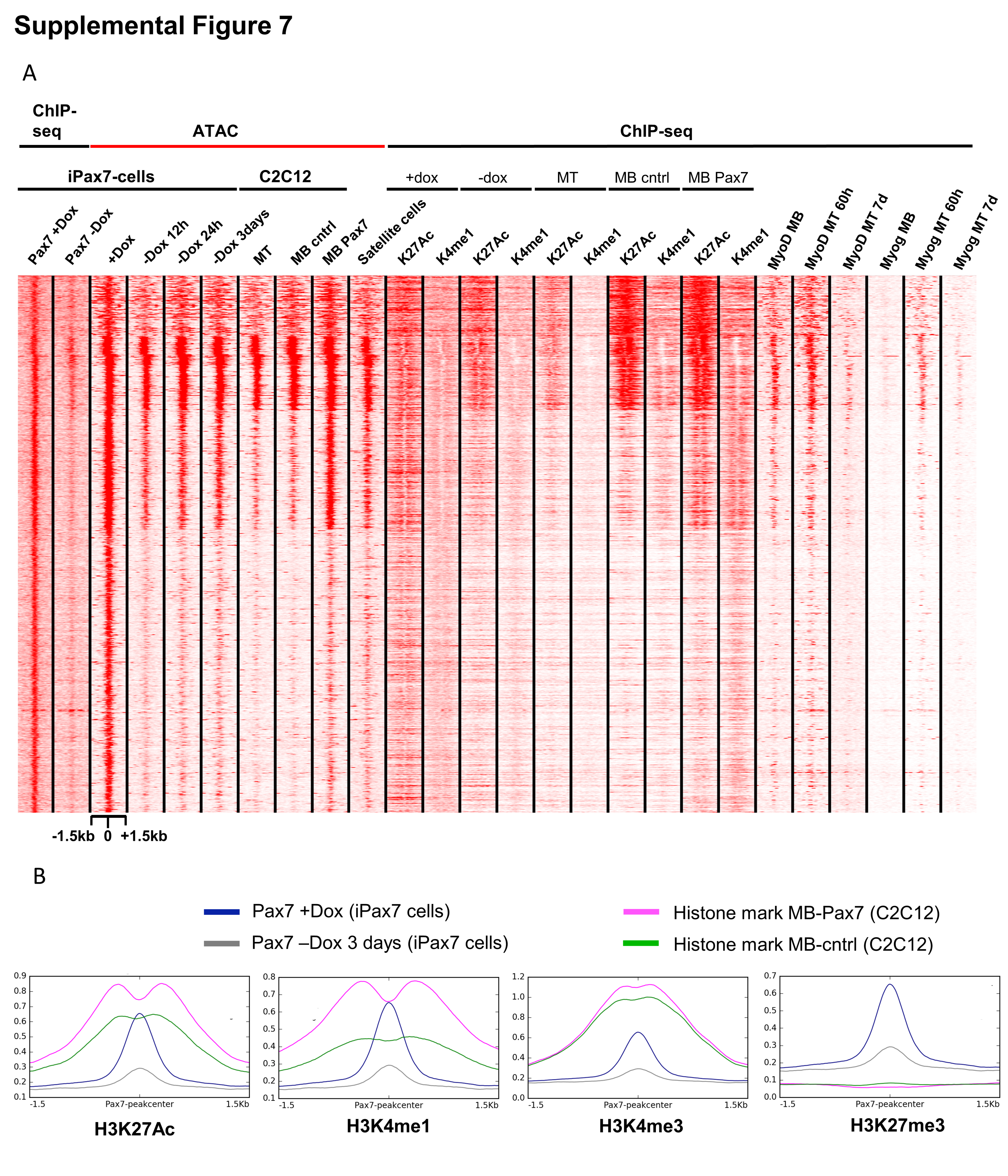

Supplement: S7 Fig — (A) Heatmap displaying acquisition of enhancer marks and increased chromatin accessibility (at Type 1 sites) after Pax7 expression in C2C12 myoblasts. ATAC-seq and ChIP-seq data are shown for iPax7 cells with or without Dox (3d) and for C2C12 with (MB Pax7) or without (MB Cntrl) Pax7 expression. Pax7 bound regions gaining an enhancer signature in C2C12 cells are not universally pre-bound by MyoD nor Myog. MB and MT, myoblasts and myotubes, respectively. (B) Metagene analysis showing the average ChIP-seq signal for Pax7 (iPax7 +Dox vs -Dox) and indicated epigenetic marks (H3K27Ac, H3K4me1, H3K4me3, H3K27me3) in C2C12 myoblasts over-expressing Pax7 (MB-Pax7) or Flag-only control (MB-cntrl) C2C12 myoblasts plotted for regions 1.5 kb upstream and downstream of the center of Pax7 binding sites. Levels of enhancer marks and bimodality increased upon introducing Pax7 in C2C12 myoblasts. (TIF) [file pone.0176190.s007.tif]
