# Supplementary material for: Pax7 remodels the chromatin landscape in skeletal muscle stem cells
Source: PLoS One. 2017 Apr 25;12(4):e0176190. doi: 10.1371/journal.pone.0176190 (PMC5404880; doi:10.1371/journal.pone.0176190)
Supplement: S1 Text — (PDF) [file pone.0176190.s010.pdf]

Supplemental Materials and Methods

RNA-seq

RNA-seq libraries (GSE89977) were prepared using SMART-Seq Ultra Low Input RNA Kit (Clontech) with the manufacturer's instructions. Libraries were sequenced (51 bp, paired end) on an Illumina HiSeq2500 machine. Raw reads were first trimmed of the first 6 bp using Trimmomatic v0.32 (1) and filtered through Bowtie2 v2.2 using default options to remove ribosomal RNAs. The remaining reads were aligned to known *Mus musculus* mm9 RefSeq genes with the TopHat2 v2.0 and Bowtie2 v2.2 tools (2) using options '--no-coverage-search --prefilter-multihits --no-discordant --no-mixed -N 0 --b2-very-sensitive'. Reads were counted across each gene with HTSeq v0.6 (3) using the default options for non-stranded, paired-end reads. The subsequent counts were normalized for each replicate using the R package DESeq2 (4), and the normalized replicates were then averaged together.

To calculate fold-change in expression, (1) genes with non-zero, non-normalized expression values (from HTSeq) in at least two replicates each from satellite cells and iPax7 (+Dox and -Dox) cells were selected and (2) the corresponding average normalized expression values (from DESeq) were used. Pearson correlation calculations were performed with the R function cor using the normalized expression of genes that have a combined non-normalized read count of at least 3 (across all conditions and cell types).

All ChIP-seq, ATAC-seq, and RNA-seq data have been submitted to the Gene Expression Omnibus (GEO, accession number GSE89977).

Gene ontology analysis

For ChIP-seq, Pax7 sites were annotated to the nearest TSS, and this gene list was used for Gene Ontology (GO) term enrichment analysis using DAVID (5, 6). Redundant groups were removed from the list and sorted by $-\log_{10}$ of the p-value assigned by DAVID. For RNA-seq, genes shown to be expressed in satellite cells and upregulated or downregulated by at least two-fold were used for GO term enrichment analysis using DAVID.

Principal component analysis

Principal component analysis was performed using the multiBigwigSummary tool of deepTools version 2.2.3 (7). All ATAC regions called by MACS2 in all samples were collectively considered for PCA. In analyses examining Pax7-bound sites, ATAC regions also bound by Pax7 were considered for PCA. Output from multiBigwigSummary tool was subsequently used to generate PCA plots using the plotPCA tool of deepTools.

***k*-means clustering and heat map generation**

For each analysis, an equal number of reads was randomly selected from each experiment. Reads within a 3 or 6 kb window around each RefSeq Transcriptional Start Sites (TSS) or center of a Pax7-bound region were collected. These regions were then clustered and visualized with a +/- 1.5 kb window around each given Pax7 peak-center as a reference using SeqMINER version 1.3.

Western blotting

Cells were collected, washed with ice-cold PBS, and lysed with lysis buffer (50 mM HEPES pH 7.0, 150 mM NaCl, 5 mM EDTA, 0.1% NP-40, 1 mM dithiothreitol (DTT) and protease and phosphatase inhibitors (aprotinin, leupeptin, pepstatin A, 4-(2-aminoethyl) benzenesulfonyl

fluoride hydrochloride (AEBSF), sodium fluoride and β-glycerophosphate)) for 30 min at 4 °C. The whole cell lysate was then spun at 14,000 rpm for 10 min at 4 °C, and the resulting supernatant was collected and analyzed by immuno-blotting.

DNA sequence motif Analysis

To search for motifs near Pax7 binding sites, we converted bed files to fasta files with sequences 250 bp upstream and 250 bp downstream of the peak center of Pax7 binding sites. Bed files were converted to fasta-format using the UCSC table browser, where repetitive sequences were masked with N, sequences were used for discovery of DNA sequence motifs using MEME, and transcription factor binding motifs were extracted from CentriMo (8, 9).

Antibodies used in this study

Antibodies directed against the following proteins and modified histones were used: MHC (Developmental Studies Hybridoma Bank (DSHB) MF20, 1:500 for Western blot, 1:20 for immunofluorescence), Pax7 (DSHB, 1:500 for Western blot, 1:200 for immunofluorescence and 3 μg for ChIP), Myf5 (Santa Cruz C-20 sc-302, 1:1000 for Western blot, 1:500 for immunofluorescence), Myogenin (DSHB F5D, 1:500 for Western blot, Rb SC576 1:100 for Immunoflourescence), α-tubulin (Sigma T5168, 1:5000 for Western blot), H3K4me1 (Abcam ab8895, 2.5 μg for ChIP), H3K4me3 (Active Motif 39159, 2.5 μg for ChIP), H3K27Ac (Abcam ab4729, 2.5 μg for ChIP), and H3K27me3 (Millipore 07-449, 2.5 μg for ChIP).

1. Bolger AM, Lohse M, Usadel B. Trimmomatic: a flexible trimmer for Illumina sequence data. Bioinformatics (Oxford, England). 2014;30(15):2114-20.

2. Kim D, Pertea G, Trapnell C, Pimentel H, Kelley R, Salzberg SL. TopHat2: accurate alignment of transcriptomes in the presence of insertions, deletions and gene fusions. Genome biology. 2013;14(4):R36.
3. Anders S, Pyl PT, Huber W. HTSeq--a Python framework to work with high-throughput sequencing data. Bioinformatics (Oxford, England). 2015;31(2):166-9.
4. Love MI, Huber W, Anders S. Moderated estimation of fold change and dispersion for RNA-seq data with DESeq2. Genome biology. 2014;15(12):550.
5. Huang da W, Sherman BT, Lempicki RA. Systematic and integrative analysis of large gene lists using DAVID bioinformatics resources. Nature protocols. 2009;4(1):44-57.
6. Huang da W, Sherman BT, Lempicki RA. Bioinformatics enrichment tools: paths toward the comprehensive functional analysis of large gene lists. Nucleic acids research. 2009;37(1):1-13.
7. Ramirez F, Ryan DP, Gruning B, Bhardwaj V, Kilpert F, Richter AS, et al. deepTools2: a next generation web server for deep-sequencing data analysis. Nucleic acids research. 2016;44(W1):W160-5.
8. Bailey TL, Machanick P. Inferring direct DNA binding from ChIP-seq. Nucleic acids research. 2012;40(17):e128.
9. Machanick P, Bailey TL. MEME-ChIP: motif analysis of large DNA datasets. Bioinformatics (Oxford, England). 2011;27(12):1696-7.
